# Unequal consequences of Covid 19: representative evidence from six countries

**DOI:** 10.1007/s11150-021-09560-z

**Published:** 2021-04-07

**Authors:** Michèle Belot, Syngjoo Choi, Egon Tripodi, Eline van den Broek-Altenburg, Julian C. Jamison, Nicholas W. Papageorge

**Affiliations:** 1 Department of Economics, Cornell University, Ithaca, USA; 2 Department of Economics, Seoul National University, Seoul, South Korea; 3 Department of Economics, University of Essex, Colchester, England; 4 College of Medicine Burlington, University of Vermont, Burlington, VT USA; 5 Department of Economics, University of Exeter, Exeter, England; 6 Department of Economics, John Hopkins University, Baltimore, MD 21218 USA

**Keywords:** Covid, Inequalities, Age, Socio-economic gradient, Mental health, Public Support, H0, H3, I1, I3, J0

## Abstract

Covid-19 and the measures taken to contain it have led to unprecedented constraints on work and leisure activities, across the world. This paper uses nationally representative surveys to document how people of different ages and incomes have been affected in the early phase of the pandemic. The data was collected in six countries (China, South Korea, Japan, Italy, UK, and US) in the third week of April 2020. First, we document changes in job circumstances and social activities. Second, we document self-reported negative and positive consequences of the crisis on well-being. We find that young people have experienced more drastic changes to their life and have been most affected economically and psychologically. There is less of a systematic pattern across income groups. While lower income groups have been more affected economically, higher income groups have experienced more changes in their social life and spending. A large fraction of people of low and high income groups report negative effects on well-being.

## Introduction

The Covid-19 pandemic has affected almost all countries in the world and has led to unprecedented measures being implemented to contain the virus. Countries have differed in their response to the epidemic. Some adopted stringent measures, such as shelter-in-place order, while others implemented early and widespread testing and tracing procedures.

The adjustments required to contain the epidemic have had a dramatic impact on how we live, our ability to work and our social life. As a consequence, the societal changes may have large repercussions both in economic and psychological terms. A key concern is that the groups that have been affected most by the measures taken are not the ones who face the highest risks of severe illness. Such misalignment between personal incentives and burdens on the one hand, and public health concerns on the other hand, are a main challenge in devising and implementing effective public policies.

In this study, we are interested in understanding better how different groups of the population have been affected by the crisis. The pandemic and the restrictions in place have affected two distinct but key sets of variables affecting well-being: economic variables, and social interactions. It is well-known that job loss for example is associated with negative mental health effects, and mental health effects often depend on how one fares relative to others (Clark (2003)). But it is also well-known that social interactions and loneliness is a major predictor of unhappiness, see for example Diener and Seligman (2002). In Maslow’s hierarchy of needs, love and belonging needs (friendship), come right after food, clothing and job safety.

Our paper is the first to document more systematically changes in economic *and* non-economic circumstances. Our study is also one of the few presenting cross-country evidence. The data consists of a sample of around 6000 individuals from three Western countries—US, UK and Italy—and three Asian countries—China, Japan and South Korea (Belot et al., 2020).

The data we use relate to the *early* phase of the pandemic (third week of April 2020) in the world. At that point, many countries had implemented drastic containment measures restricting mobility and social interactions. The samples are nationally representative on three dimensions: age, gender and income, as well as on race in the US. We did not require representativeness on geographical location, but as shown in Table A7, our sample is well distributed across various regions in each country. The data was collected in the third week of April 2020, at a point where most countries had introduced some form of restrictions.

Our study documents the effects of the crisis using three sets of variables. First, we document objective changes in economic circumstances. In particular, we examine whether people lost their job specifically because of the crisis (temporarily or permanently), whether they started working from home and how their consumption expenditures changed relative to January 2020. Second, we document objective changes in social activities. We look at the frequency of different leisure activities that have a social component—attending large social gatherings, visiting family and friends, and going to large close or open public spaces. Participants were asked to indicate at which frequency they were engaging in these activities before the pandemic. Finally, we examine self-reported effects on individual well-being. Negative effects we consider include boredom, loneliness, trouble sleeping, anxiety and stress, and conflicts with friends and neighbors. These negative, non-financial effects are potentially important because they speak to the burden of complying with measures to contain the pandemic and also shape incentives for individuals to follow social distance measures. We also consider potential positive effects, such as spending more time with family, enjoying more free time, and reductions in pollution and noise.

At the time of data collection, the countries we examined were at different phases of the epidemic and had implemented different measures.^1^ These differences, on top of differences in other factors (such as cultural attitudes), can all contribute to explain the cross-country differences in the nature of the Covid-19 effects. Instead of identifying the causes of such differences, this paper marks a first step in understanding how the pandemic has affected different age and income groups across countries. We will refrain from speculating on the causal relationships.

Our work complements preliminary evidence put forward in a few contemporaneous studies focusing on specific countries and specific aspects of the crisis such as Adams et al. (2020), Montenovo et al. (2020), Fairlie et al. (2020), von Gaudecker et al. (2020). Existing studies have documented large discrepancies in the economic impact of the crisis across socio-economic groups, with the least advantaged being more likely to be negatively affected. There is also evidence that these groups may have been more adversely affected psychologically (Witteveen and Velthorst, 2020). We contribute here by broadening the set of variables we consider (covering both economic and non-economic circumstances), and we are the first to present comparable data covering Western and Asian countries.

The information contained in this survey is self-reported and, although it is built to be representative on key socio-demographics, individuals self-select in answering the survey. Relative to administrative data, surveys are subject to several types of reporting biases (such as recall bias). Despite these limitations, our data offers timely evidence that serves as crucial input for model calibration (e.g., Brotherhood et al., 2020, Manzo and van de Rijt, 2020) and policy discussions (e.g., Acemoglu et al., 2020, Dowd et al., 2020, Ichino et al., 2020). Our survey allows to relate a rich set of individual characteristics to important outcomes that would not be possible to link at the individual level from existing surveys. Cross-country comparisons from nationally representative samples help highlight systematic patterns and should be used in conjunction with other empirical evidence to better understand how different policies in different countries have contributed to contain the pandemic, as well as its economic impacts.

The remaining of the paper is structured as follows. In Section *Data and Empirical Strategy* we present our data and empirical strategy, in Section *Results* we present the results and in Section *Discussion*, we discuss the implications of our findings for policy.

## Data and empirical strategy

### Data

We use data that was collected by Belot et al. (2020) between April 15 and April 23.^2^ This dataset includes 6082 respondents; roughly 1000 from each of the six countries. Three Asian countries (China, Japan and South Korea) and three western countries (Italy, the United Kingdom, and the United States). For each country, the sample is nationally representative along age, gender, and (pre-Covid) household income. In the United States the data includes respondents from the 4 most populous states: California, Florida, New York, and Texas. American respondents self-identify their race, and the sample is also nationally representative along this dimension.

Data were collected between April 15 and April 23 through market research firms Lucid (for Italy, UK, and US) and dataSpring (for China, Japan, and Korea). Potential participants were drawn from several different panels to which the surveying firms have access. Invitations to participate in the online survey (programmed in Qualtrics) were sent via email, with new invitations being sent daily up to the point where representativeness along relevant dimensions was achieved. Before participating in the survey respondents review a consent form that specifies that individual-level data will be made publicly available in anonymized form (excluding a short list of health related variables clearly marked in the survey). The ethics board at the University of Exeter approved this study prior to data collection.

We took several steps to ensure data quality. We gave monetary compensation to respondents for their participation, following general compensation schemes defined by our partner surveying firms–which depend on estimated survey completion time. The median time to complete the survey was about 14 min. Respondents were prevented from taking the survey multiple times, and they were excluded for completing the survey too quickly (in under 50% of the median response time).

The data include basic socio-demographic, household, and health status information, exposure to the disease at work and living conditions, economic and psychological impacts of the pandemic, beliefs, and attitudes toward the government’s response.^3^ In this paper, we focus the main economic and non-financial impacts of the pandemic to document the gradients along the main socio-demographics, and compare them across countries.^4^

Relative to most existing datasets, this survey presents several advantages but also limitations. First, all this data is self-reported. Relative to administrative data, this data is subject to reporting biases. We are not aware of existing administrative datasets that would allow to address the research questions of this paper; many of the outcomes we are interested in (such as psychological well-being and social interactions) would be very expensive to track with independent measurements at such scale and would be hard to link to other administrative datasets. Second, an important strength of this survey is that it provides internationally comparable cross-country data that links a reach set of individual characteristics, circumstances and outcomes. Still, the reader should interpret cross-country comparisons with caution, as they may stem from how policies differed across countries, but also from cultural and institutional differences, as well as the stage of the pandemic different countries were experiencing at the time of data collection. Third, this data was collected only at one point in time, at the beginning of the pandemic. Existing surveys that are ongoing will allow researchers to observe changes in behavior during and after the pandemic relative to “normal” times. To partly counter this limitation, our survey collects retrospective information and asks specifically about changes in behavior. An advantage of this information is that we can ask questions that are pertinent to the current pandemic. At the same time, one should be concerned about how recall bias may affect the results.^5^ Finally, while national representativeness on socio-demographics of our dataset constitutes a marked improvement over surveys with convenience samples, we caution that online recruitment respondents may feature selection on unobservable characteristics.^6^

### Methods

Our analysis is based on ordinary least square models (or linear probability models when the outcome is binary). The right hand side variables include age and income dummies, as well as additional control variables such as gender, a rural-urban indicator, and regional dummies. The age categories we consider are: below 25 (between 18 and 25), 26–45, 46–65 and above 65. For income, we use the categorical variable indicating the household income quintile as reported by the respondent.^7^

Despite having some information on location, samples are too small to focus our study of unequal consequences of Covid 19 at subnational level. In *Geographical variation*, we come back to the issue of sub-national heterogeneity in response, by presenting our analyses separately for regions with a high prevalence of Covid 19 and those with a low prevalence.

Unless otherwise stated we include the full sample in the analyses. We will interpret significance levels of the coefficients at face value, without implementing corrections for multiple hypotheses testing. Given how little is known on the topic this analysis is necessarily exploratory, and confirmatory research will be needed. Yet, the goal is to see if a coherent story emerges.

## Results

We first document the objective changes in people’s life circumstances, focusing on work, consumption and social activities. Second, we examine self-reported effects on well-being, which reflect additional non-financial consequences that respondents attribute to the crisis. Finally, we comment on how the patterns described vary across geographical location, specifically according to the local prevalence of the disease as recorded in the third week of April.

### Objective changes in work, consumption and social activities

#### Impact on economic circumstances

We first examine how likely it is that people lost their job as a consequence of the crisis (temporarily or permanently), and whether they have started teleworking. We then look at reported changes in spending, relative to the month of January 2020.

Note that countries have differed in the schemes they have implemented to mitigate the impact of the crisis in the labor market. Some of them implemented transfer schemes, others have implemented policies to limit dismissals (see Gentilini et al., 2020).

In order to visualize more directly age and income differences, here and after, we present figures showing the estimates of gender, age and income dummies. The reference groups will always be those below age 26 and those in the lowest income quintile respectively. Below each figure, we also report the overall mean of the outcome of interest. The full results are presented in Tables in the Appendix, to which we will also refer.

**Fig. 1:**
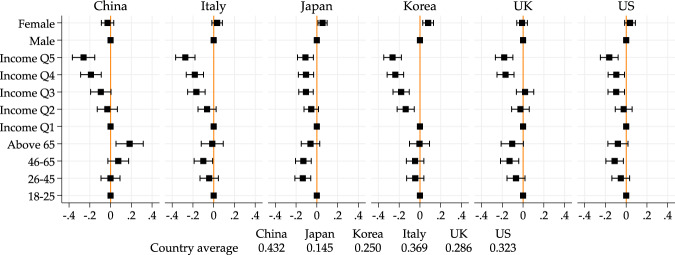
Age and income gradients on losing job at least temporarily. *Note*: Point estimates and 95% confidence intervals from a linear regression model of job loss (permanent or temporary) on income quintile, age group, gender, geographical controls, and employment status pre-pandemic. Figure based on regression results in Table A1. Male, 18–25 and Income Q1 are baseline categories for gender, age and income quintile groups, respectively. Country averages of the outcome variables used for the regression are reported below the figure

Figure 1 plots the age and income dummies for the probability of having lost one’s job at least temporarily (Table A1 shows the full results). The first observation is that there are substantial differences in means across countries. In Japan, 85% of people report no change. At the other extreme, in China, 43% report having lost their job at least temporarily.

Looking at differences across income groups, we find a remarkably similar pattern across all countries, with higher income groups being substantially less likely to have lost their job. Comparing the fifth quintile to the first quintile, the differences range from 10 percentage points in Japan to 27 percentage points in Italy.

Differences across age groups are less marked overall. There are significant differences in Western countries and in Japan, where the middle-aged group (46–65) has been least affected. In Korea, we find no evidence of a significant age gradient and in China, we find that the older group has been substantially *more* affected. The above 66 are 18 percentage points more likely to have lost their job than those below age 26.

Note that we do not see significant differences in the probability of job loss according to gender, except for Japan and Korean, where women have been more affected.

**Fig. 2:**
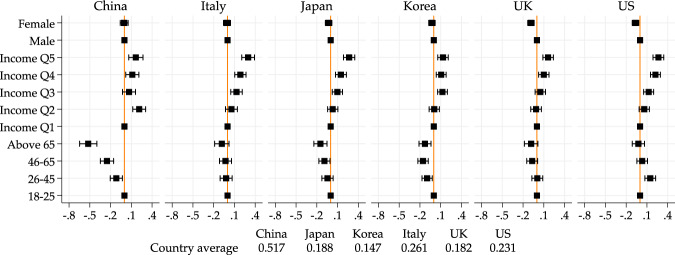
Age and income gradients on teleworking. *Note*: Point estimates and 95% confidence intervals from a linear regression model of government support on income quintile, age group, gender, geographical controls, and employment status pre-pandemic. Figure based on regression results in Table A2. Male, 18–25 and Income Q1 are baseline categories for gender, age and income quintile groups, respectively. Country averages of the outcome variables used for the regression are reported below the figure

One key factor determining whether people were able to keep their job or not was the ability to work from home. In Fig. 2 and Table A2, we examine the probability of having started teleworking. Here in all countries, we see a very similar pattern in the probability of teleworking: Younger groups and higher income groups are substantially more likely to be teleworking than those in the bottom 20% income. In China, the 46–65 are 26 percentage points less likely to telework relative to to the 18–25 group. The difference is smaller but remains large in other countries, except Italy, where there is no significant difference, and the US, where the 26–45 is more likely to telework than the below age 26.

There is less of a consistent pattern across countries for gender: in the UK and the US, women are less likely to have started teleworking but the gender differences are close to zero and insignificant in the four other countries.

**Fig. 3:**
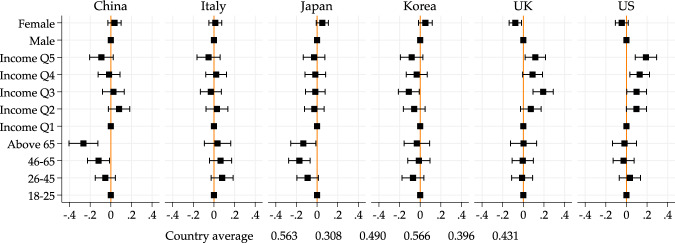
Age and income gradients on drop in household spending. *Note*: Point estimates and 95% confidence intervals from a linear probability model of an indicator variable. This indicator denotes whether respondents reports a drop in household consumption. Covariates include income quintile, age group, gender, geographical controls and employment status pre-pandemic. Figure based on regression results in Table A3. Male, 18–25 and Income Q1 are baseline categories for gender, age and income quintile groups, respectively. Country average of the outcome variables used for the regression are reported below the figure

Finally, we look at reported changes in spending relative to the month of January 2020 (Fig. 3 and Table A3). While people may have experienced drops in income due to the crisis, the measures implemented in most countries also limited opportunities for consumption. At the point of data collection, most street shops, leisure venues (such as cinemas and theaters) and restaurants were closed in Italy, the UK and the US. Here we find interesting differences across countries: In China and Japan, older groups reduced their spending more than younger groups. We see no such difference in the Western countries and Korea. In the UK and the US, the higher income groups are more likely to report a drop in spending than lower income groups. This echoes evidence from credit card transaction data reported in Chetty et al. (2020) for the US. Differences according to gender are again less marked and not systematic across countries. In the UK, women are less likely to report a drop, in Japan, it is the reverse. In all other countries, the differences across gender are not statistically significant.

Despite the diversity of measures implemented across countries, we see interesting systematic patterns emerging: Perhaps not surprisingly, younger people and lower income groups have been economically more affected in the labor market. Gender differences are, however, less systematic across countries.

#### Impact on leisure and social activities

Besides having a substantial effect on work, the crisis also substantially affected the private sphere of life. To assess the impact on social life and leisure, we construct an index measuring the degree of engagement in different leisure activities that have a social component. Respondents were asked to indicate the frequency of engagement in a series of activities at different points in time: in normal times before the outbreak, at the start of the outbreak and at the time of the survey. The index aggregates information from four variables: participation in large social gatherings, going to large close spaces (such as a museum or a shopping center), going to large open spaces (such as a public park), visiting friends/family.

**Fig. 4:**
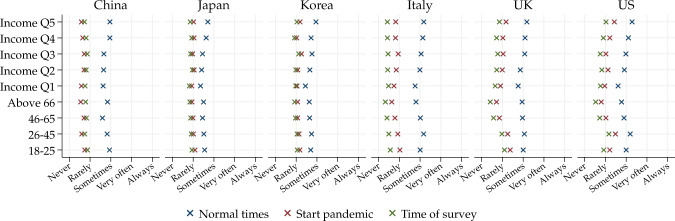
Social interactions over time, by age and income groups. *Note:* We report group averages of an index that includes frequency of (i) participation in large social gatherings, (ii) visit to large open spaces, (iii) large close spaces, and (iv) visits to friends or family. The index is constructed by averaging (i)–(iv) frequencies on a 1–5 scale, where “1” is “Never” and “5” is “Always”

Figure 4 shows the mean reported levels of our index variable at three points in time—in normal times before the outbreak, at the start of the outbreak and at the time of the survey. In all countries, the younger groups (18–25 or 26–45) are most engaged in social activities. But the older groups appear to have reduced their social life most. There is also a clear income gradient: Higher income groups are more likely to engage in leisure activities with a social component, in all countries. Since those were effectively discouraged or forbidden at the time of the survey, higher income groups by then had experienced a larger negative impact on their social life in most of the countries. This is evidenced by marked income gradients on how bothered they report being for not being able to participate in large social gatherings, go to large (close or open) spaces, and visit friends or family (see Table A6).

Summarizing and looking across countries, we find that those who experienced the largest negative economic impacts are the young, while older groups and high income groups experienced the largest negative impact in their social life and leisure.

### Self-reported impact on well-being

We now turn to the self-reported impact of the pandemic (and measures implemented) on well-being. The question asked refers specifically to changes experienced as a consequence of the epidemic.^8^ Negative effects include anxiety, trouble sleeping, increased conflicts, boredom or loneliness. Positive effects include more time with family, more free time, less pollution or less noise. Survey participants could indicate as many as applicable. We construct two simple variables counting the number of positive and negative effects indicated, respectively.

**Fig. 5:**
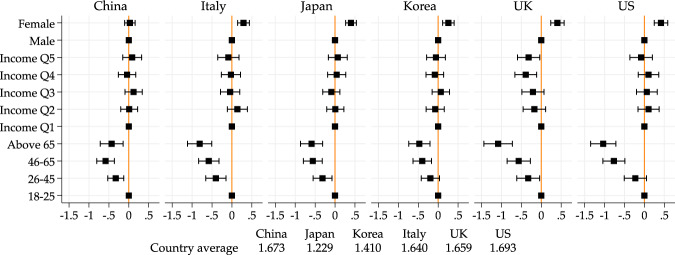
Age and income gradients on negative well-being. *Note*: Point estimates and 95% confidence intervals from a linear regression model of number of negative well-being consequences of the pandemic (which include: (i) boredom, (ii) loneliness, (iii) trouble sleeping, (iv) general anxiety and stress, and (v) increased conflicts with friends/family/neighbors) on income quintile, age group, gender and geographical controls. Figure based on regression results in Table A4. Male, 18–25 and Income Q1 are baseline categories for gender, age and income quintile groups, respectively. Country averages of the outcome variable used for the regression are reported below the figure

Figure 5 and Table A4 present the evidence on negative effects. We find that younger people are substantially more likely to report negative effects, in all countries. This is perhaps not surprising because younger people report larger changes in their life, but on the other hand, one could have expected older people to experience more psychological distress as they are in a higher risk group of suffering from the disease.

Understanding the higher psychological costs of the younger groups is important because they may comply less with social distancing measures. Here the evidence suggests that the objective changes in people’s lives (more prevalent among younger groups) have a larger psychological impact than the threat of the disease.

**Fig. 6:**
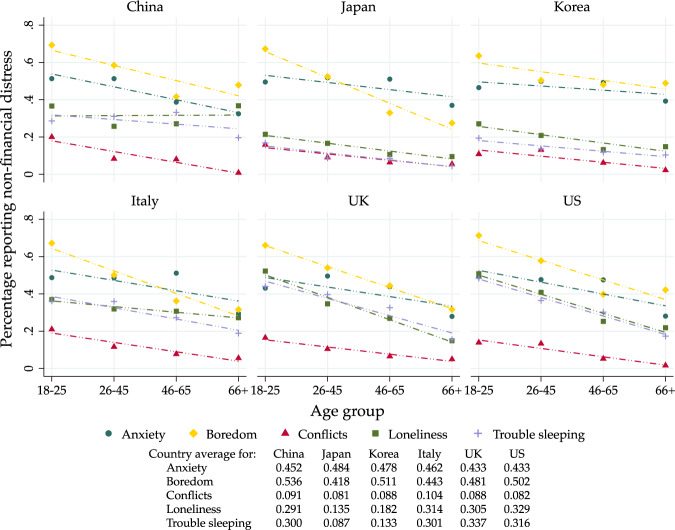
Age gradients on negative well-being. *Note*: We report group averages for the probability of experiencing each one of the negative well-being effects we asked about: Anxiety, Boredom, Conflict with friends/family/neighbors, Loneliness and Trouble sleeping. Dashed lines represent linear fits. Country averages of the outcome variable are reported below the figure

Figure 6 examines age differences in each of the dimensions considered. We find that younger people are more likely to report each of the suggested negative effect, with boredom and anxiety being the more prevalent ones in all countries.

Differences across income groups are much less clear (see Table A4): It is only in the UK that we see that higher income groups report less negative effects on well-being. In all other countries, the differences across income groups are smaller and not statistically significant.

Gender, on the other hand, is now a clear predictor of negative well-being effects. Across all countries (except for China), women are more likely to report such effects.

We also find that people report experiencing some benefits from the pandemic—between enjoying more free time, enjoying time with family, cleaner air, and less noise pollution (Table A5). There is no age gradient in Japan, Italy or the US, but there is one in China, and Korea, with older groups reporting less positive effects. The UK is the only country where respondents in the oldest age group report more positive effects.

The pattern is also less clear across countries according to income. The overall evidence points in the direction of higher income groups reporting more positive effects, but the effects are not significant in all countries and in the UK, the middle income groups report less positive effects than the lowest income quintile.

Finally, we see no significant gender difference in the probability of reporting positive effects.

Summarizing, we find that young people are most negatively affected in non-financial, psychological terms; positive effects are less clearly differentiated across age and income groups.

### Geographical variation

As mentioned earlier, we did not aim for geographical representativeness. Nevertheless, since our samples are well distributed across the various regions in each country (see Table A7), it is possible to examine whether the patterns we identified differ across regions depending on how severely they have been affected by the epidemic (at that point in time). To do this, we sort regions in each country in two categories: low or high prevalence, depending on whether the local (regional) prevalence rate was above or below the median prevalence rate in the country. Prevalence rates are measured as the number of positive tests per capita in the 3 weeks prior to the survey (from March 25 to April 15).^9^ Of course there are clear potential issues of measurement error, but we would argue that these are unlikely to affect the ranking of regions within a country.

In Table A9 we report the means for each outcome discussed above. We only find within-country differences between high and low prevalence areas for the probability of teleworking. All other outcomes, whether they relate to objective changes in economic circumstances, leisure and social activities or well-being show little variation according to prevalence. In China, we observe that the probability of job loss is higher in areas with low prevalence of the disease, perhaps because they were at the time areas that are richer and densely populated.

In Table A10 we report estimates of linear age and income gradients for each subsample (low and high prevalence). Overall the patterns we described above hold. There are a few exceptions, but it is clear that the patterns are very similar across regions within the same country, and contrast with the differences observed across countries.

The lack of variation across regions is maybe not surprising given that most countries had implemented measures at the nationwide level. But even in countries where measures were determined at a more regional level (such as in the US), we do not find large differences in the patterns of impact of the crisis.

## Discussion

The pandemic and the measures taken in response to it across the world appear to have affected different groups of the population in different ways. As a result, some subgroups of the population are economically and psychologically more vulnerable than other subgroups. The evidence presented here is purely descriptive. The complexity and diversity of measures implemented challenges the identification of mechanisms that explain the outcomes and we deliberately refrained from making causal statements or speculating on the reasons why observe such heterogeneity.

Understanding the heterogeneous nature of the impact of Covid-19 is however a necessary step toward improving the current set of policy tools, as the impact is likely to affect compliance with measures that align with societal goals of containing the pandemic while minimizing economic and social damage.^10^

Our study contributes to the existing literature by presenting a cross-country comparison, including Western and Asian countries, and by presenting broader evidence on how people’s lives have been affected. While the economic consequences of the crisis have by now been well documented, much less emphasis has been given to objective changes in other aspects of life. We also examine effects on well-being, which have been the subject of several studies. Our contribution here is to be able to link those to a broader set changes in circumstances and to include both positive and negative effects on well-being.

In the six countries we surveyed, we find consistent evidence that younger people have been more negatively affected—both economically and psychologically. At the time of data collection, all countries were facing some of form of restrictions, but these restrictions differed in strength. The remarkable similarity of age gradients across countries is our view a striking and important fact.

There is a less clear pattern in how people of different groups have been affected by income. Perhaps this is because all income groups have experienced substantial changes in their lives: Lower income groups have also experienced more changes in their labor market circumstances, higher income groups have experienced more changes in their social and leisure activities. However, our findings on income groups are not definitive because the extent of economic impacts was not fully revealed by the time of our survey.

Here we show that lower income groups have suffered more in economic terms, but we also show that higher income groups have experienced larger changes in their private/social life. These facts could potentially explain why we do not find clear income gradients in negative psychological effects, which one may have expected to find.

This evidence that younger people are more affected by the pandemic strengthens the case for more differentiated policies that shield the young from the negative consequences of the epidemic and necessary measures. A number of recent papers propose policies that target the older part of the population (see, for the effects of age-specific policies, Acemoglu et al., 2020, Brotherhood et al., 2020, Favero et al., 2020). The advantage would be that such targeted policies is to mitigate the economic costs of the crisis, while shielding those with the highest health risks. However, the consequences of shutting down interactions between the old and the young are not yet well understood. People from different age groups rely on each other for many reasons, and breaking such inter-generational bonds and arrangements may have negative consequences, which are difficult to assess and will require more research. It is also imperative to find ways to match young people’s incentives and burdens of complying to public policies.

Finally, our data shows notable heterogeneities in how women have been affected across countries. We corroborate previous evidence showing that women are less likely to have started teleworking in the UK and the US. These findings echo evidence presented by Alon et al. (2020) who show that women are concentrated in sectors disproportionately affected by the crisis^11^. However, we see little gender differences in the other countries we surveyed. The most clear and systematic evidence of a gender gap is in our measure of negative well-being due to the crisis, with women being much more likely to report those in all countries but China. Potential explanations for this finding may be found in the widening gender gap in the allocation of household tasks (e.g., Biroli et al., 2020, Daniela, Oggero, Profeta and Rossi, 2020), or in the increased incidence of conflicts and domestic violence (e.g., Hsu and Henke, 2020, Leslie and Wilson, 2020).

## Supplementary information

Online Supplementary Information

Supplementary figures

## Footnotes

1 See for the six countries, in Figs. A1 and A2 of the Online Supplementary Material, how the data collection time window overlaps with the time series of the number of confirmed infected people and deaths per million inhabitants together with the stringency index of government policies to contain the pandemic, based on the Oxford Covid-19 Government Response Tracker data (Hale et al., 2020).

2 Made available as a public good on May 4, 2020 (https://osf.io/aubkc/).

3 The survey is conducted at the individual level. Some questions (such as household income) capture information about other household members, which subjects may observe with noise. All such questions ask about household (rather than individual) quantities, so to avoid social desirability biases due to social norms on intra-household comparisons (Murray-Close and Heggeness, 2019).

4 In a companion paper, we study the determinants of self-protective behavior in the United States (Papageorge et al., 2020). Others have used our dataset to study e.g., gender inequality in economic consequences of the pandemic (Dang et al., 2020), age gradients in health-risk perceptions (Bordalo et al., 2020), and calibrate models of disease spread (Brotherhood et al., 2020, Manzo and van de Rijt, 2020).

5 Some of our questions ask respondents about changes in outcomes relative to January 2020 or relative to “normal times”. Relative to the time of data collection, such questions require recall of events that happened in a relatively long time window, 3 months before. The literature on recall bias gives limited guidance on how to optimize survey design. However, we think recall bias is unlikely to systematically drive our results for at least three reasons. First, asking about events that happened 3 months ago does not necessarily induce more recall bias than for more recent events. Theoretically, recall can be hard for long time windows that include distant events (due to *omission*), but can also be hard for short time windows when events are recent (due to *telescoping*, i.e., the tendency to misattribute the timing of events) (Sudman and Bradburn, 1973). A recent empirical paper finds that *telescoping* is the prevalent force in patient recall of doctor visits (Kim, Li, Scott and Clarke, 2018). Second, recall bias should be more severe for questions that elicit precise quantities with narrow time stamps, and Hiroyuki et al. (2010) offer evidence consistent with that view. Third, if our data captures a gradient in some country but not in others, for this result to be driven by recall bias one would need a story by which recall bias differs across countries. With our data, we can compare some of our results to evidence from other studies that use administrative data. We come back to this in *Impact on economic circumstances*, when we discuss a study from Chetty et al. (2020), who find similar gradients in consumption drops for the U.S. (as captured by credit card transaction data).

6 Our data is nationally representative on important targeted socio-demographics and has adequate geographical representation for each country (see Table A7). However, online surveys do not capture people who do not use the internet and/or are not willing to answer online surveys.

7 For the income question, respondents choose one of five income brackets, which are obtained by calculating quintiles of the gross household income distribution from the last available wave of nationally representative household surveys (or census data), as available at the Luxembourg Income Study. We allowed a sixth category “Prefer not to answer”. See Belot et al. (2020) for actual income brackets by country.

8 The exact question is: “Have you experienced any positive (negative) non-financial effects from the societal changes occurring due to the epidemic”.

9 Table A8 summarizes sources of such disaggregated data.

10 For example, low levels of compliance among young people is a source of concern because it may increase mortality in older age groups (see e.g., Dowd et al., 2020, Fenoll and Grossbard, 2020, Harris, 2020).

11 See also Etheridge et al. (2020) for evidence from the UK.
