# Supplementary material for: Unequal consequences of Covid 19: representative evidence from six countries
Source: Rev Econ Househ. 2021 Apr 7;19(3):769–83. doi: 10.1007/s11150-021-09560-z (PMC8025452; doi:10.1007/s11150-021-09560-z)
Supplement: Supplementary file 1 — Online Supplementary Information [file 11150_2021_9560_MOESM1_ESM.html]

Replication of results for ''Unequal Consequences of Covid 19: Representative Evidence from Six Countries''

Michele Belot, Syngjoo Choi, Egon Tripodi, Eline van den Broek-Altenburg, Julian C. Jamison, Nicholas W. Papageorge


---

**DATA CLEANING**

```
      . import delimited "data_for_replication/FullTS.csv", encoding(UTF-8) clear
      (10 vars, 18,798 obs)

      . gen newdate = date(date,"YMD")

      . keep if inrange(newdate,21999,22020)
      (16,774 observations deleted)

      . format newdate %td

      . collapse (sum) three_weeks_newcases = newcases, by(country prov_region)

      . rename prov_region region


      . replace country="UK" if country=="United Kingdom"
      (12 real changes made)

      . replace region ="Friuli-Venezia Giulia" if region== "Friuli V. G."
      (1 real change made)

      . replace region ="Emilia-Romagna" if region== "Emilia Romagna"
      (1 real change made)

      . replace region ="Greater London" if region== "London"
      (1 real change made)

      . replace region = "Daegu Metropolitan City" if region== "Daegu"
      (1 real change made)

      . replace region ="Ulsan Metropolitan City" if region== "Ulsan"
      (1 real change made)

      . replace region ="Incheon Metropolitan City" if region== "Incheon"
      variable region was str24 now str25
      (1 real change made)

      . replace region ="Jeju Island" if region== "Jeju-do"
      (1 real change made)

      . replace region ="Jeollabuk do" if region== "Jeollabuk-do"
      (1 real change made)

      . replace region ="Chung-cheong bukdo" if region== "Chungcheongbuk-do"
      (1 real change made)

      . replace region ="Sejong Special Self-governing City" if region=="Sejong"
      variable region was str25 now str34
      (1 real change made)

      . replace region ="Yorkshire and the Humber" if region== "Yorkshire and The Humber"
      (1 real change made)

      . replace region ="Kansai" if region== "Kansai "
      (1 real change made)

      . merge 1:1 country region using "data_for_replication/geo_distribution.dta"

          Result                           # of obs.
          -----------------------------------------
          not matched                            10
              from master                        10  (_merge==1)
              from using                          0  (_merge==2)

          matched                                82  (_merge==3)
          -----------------------------------------

      . drop if _merge!=3
      (10 observations deleted)

      . drop _merge

      . gen recent_cases_percapita = three_weeks_newcases/(population*1000000)

      . save "data_produced/recent_cases.dta", replace
      file data_produced/recent_cases.dta saved

      . use "data_for_replication/public_dataset.dta", clear

      . sdecode region, replace


      . replace region = "Fujian" if region == "Fujian " 
      (48 real changes made)

      . replace region = "Gansu" if region == "Gansu " 
      (8 real changes made)

      . replace region ="Kansai" if region== "Kansai "
      (170 real changes made)

      . merge m:m region using "data_produced/recent_cases.dta", nogen force
      (note: variable country was str5, now str14 to accommodate using data's values)

          Result                           # of obs.
          -----------------------------------------
          not matched                             0
          matched                             6,089  
          -----------------------------------------

      . gen high_prevalence=1

      . replace high_prevalence = 0 if inlist(region,"Hainan","Qinghai","Hunan","Anhui","Henan","Jiangxi") // China
      (170 real changes made)

      . replace high_prevalence = 0 if inlist(region,"Guizhou","Hubei","Gansu","Hebei","Shandong") // China
      (222 real changes made)

      . replace high_prevalence = 0 if inlist(region,"Calabria","Sicilia","Basilicata","Sardegna","Campania") // Italy
      (244 real changes made)

      . replace high_prevalence = 0 if inlist(region,"Puglia","Lazio","Molise","Umbria","Abruzzo") // Italy
      (235 real changes made)

      . replace high_prevalence = 0 if inlist(region,"Chūgoku","Tōhoku","Hokkaidō","Shikoku") // Japan
      (189 real changes made)

      . replace high_prevalence = 0 if inlist(region,"Jeollabuk do","Chung-cheong bukdo","Busan","Jeollanam-do","Ulsan Metropolitan City") // Korea
      (179 real changes made)

      . replace high_prevalence = 0 if inlist(region,"Gyeongsangnam-do","Chungcheongnam-do","Daejeon") // Korea
      (101 real changes made)

      . replace high_prevalence = 0 if inlist(region,"South West","East Midlands","East of England","Northern Ireland","Yorkshire and the Humber","South East") //UK
      (497 real changes made)

      . replace high_prevalence = 0 if inlist(region,"Texas","California") //US
      (478 real changes made)

      . sencode region, replace


      . replace country = "1" if country == "china"
      (996 real changes made)

      . replace country = "2" if country == "japan"
      (1,015 real changes made)

      . replace country = "3" if country == "korea"
      (963 real changes made)

      . replace country = "4" if country == "italy"
      (1,044 real changes made)

      . replace country = "5" if country == "uk"
      (1,016 real changes made)

      . replace country = "6" if country == "us"
      (1,055 real changes made)

      . destring country, replace
      country: all characters numeric; replaced as byte

      . lab def country 1 "China" 2 "Japan" 3 "Korea" 4 "Italy" 5 "UK" 6 "US"

      . lab val country country

      . replace work_change_due_pandemic=3 if work_change_due_pandemic ==-99 // basically saying that there was no change in work arrangement for folks that aren't working
      (938 real changes made)

      . generate age_large=.
      (6,089 missing values generated)

      . replace age_large=1 if age_group==1
      (736 real changes made)

      . replace age_large=2 if age_group==2 | age_group==3
      (2,233 real changes made)

      . replace age_large=3 if age_group==4 | age_group==5
      (2,080 real changes made)

      . replace age_large=4 if age_group==6 | age_group==7
      (1,035 real changes made)

      . label define age_large_lab 1 "18 to 25" 2 "26 to 45" 3 "46 to 65" 4 "Above 66"

      . label values age_large age_large_l

      . gen id=_n

      . bys id: egen pos_non_fin=sum(pos_nonfin_freetime+pos_nonfin_lesspolution+pos_nonfin_lessnoise+pos_nonfin_other)

      . bys id: egen neg_non_fin=sum(neg_nonfin_boredom+neg_nonfin_loneliness+neg_nonfin_troublesleep+neg_nonfin_anxiety+neg_nonfin_conflicts+neg_nonfin_other)

      . gen started_teleworking=(work_change_due_pandemic==2)

      . gen employment_status_pre_pandemic = 1 if labor_status!=4 | inlist(lost_job,1,2)
      (1,693 missing values generated)

      . replace employment_status_pre_pandemic= 0 if labor_status==4 & lost_job!=1 & lost_job!=2
      (1,693 real changes made)

      . la def employment_status_pre_pandemic 0 "Not employed before pandemic" 1 "Employed before pandemic"

      . la val employment_status_pre_pandemic employment_status_pre_pandemic

      . gen drop_savings=(inlist(change_savings,1,2))

      . gen lost_job_atleast_temporarily=(inlist(lost_job,1,2))

      . replace work_change_due_pandemic = 5 if  work_change_due_pandemic ==4
      (331 real changes made)

      . replace work_change_due_pandemic =4 if work_change_due_pandemic==3 & labor_status==4
      (1,245 real changes made)

      . la def work_change_due_pandemic_new 1 "I do not work anymore" 2 "I started teleworking" 3 "No change, employed" 4 "No change, not employed" 5 "Other"

      . la val work_change_due_pandemic work_change_due_pandemic_new

      . gen female = (gender==1)

      . pca bothered_meeting_folks bothered_leisure_activities bothered_shop_nonessentials, comp(1)

      Principal components/correlation                 Number of obs    =      6,089
                                                       Number of comp.  =          1
                                                       Trace            =          3
          Rotation: (unrotated = principal)            Rho              =     0.7634

          --------------------------------------------------------------------------
             Component |   Eigenvalue   Difference         Proportion   Cumulative
          -------------+------------------------------------------------------------
                 Comp1 |      2.29014      1.87374             0.7634       0.7634
                 Comp2 |      .416399      .122942             0.1388       0.9022
                 Comp3 |      .293457            .             0.0978       1.0000
          --------------------------------------------------------------------------

      Principal components (eigenvectors) 

          --------------------------------------
              Variable |    Comp1 | Unexplained 
          -------------+----------+-------------
          bothered_m~s |   0.5792 |       .2317 
          bothered_l~s |   0.5922 |       .1969 
          bothered_s~s |   0.5602 |       .2812 
          --------------------------------------

      . predict bothered_social_distance
      (score assumed)

      Scoring coefficients 
          sum of squares(column-loading) = 1

          ------------------------
              Variable |    Comp1 
          -------------+----------
          bothered_m~s |   0.5792 
          bothered_l~s |   0.5922 
          bothered_s~s |   0.5602 
          ------------------------

      . gen fall_consumption = (inlist(change_weekly_expenses,1,2))

      . save "data_produced/clean_data", replace
      file data_produced/clean_data.dta saved
```

---

**REPLICATE FIGURE 1**

```
      . use "data_produced/clean_data", clear

      . table country, c(mean lost_job_atleast_temporarily) f(%9.3f)

      --------------------------
        country | mean(lost_j~y)
      ----------+---------------
          China |          0.432
          Japan |          0.145
          Korea |          0.250
          Italy |          0.369
             UK |          0.286
             US |          0.323
      --------------------------

      . qui reg lost_job_atleast_temporarily i.age_large i.income i.female ///
               i.current_living_area i.region i.employment_status_pre_pandemic if country==1


      . regsave using "data_produced/results", addlabel(country, China) replace
      file data_produced/results.dta saved

      . forval x=2(1)6 {
      . local country_name : label country `x'
      . qui reg lost_job_atleast_temporarily i.age_large i.income i.female ///
               i.current_living_area i.region i.employment_status_pre_pandemic if country==`x'

      . regsave using "data_produced/results", addlabel(country, `country_name') append
      . }
      file data_produced/results.dta saved
      file data_produced/results.dta saved
      file data_produced/results.dta saved
      (note: variable country was str2, now str5 to accommodate using data's values)
      file data_produced/results.dta saved
      (note: variable country was str2, now str5 to accommodate using data's values)
      file data_produced/results.dta saved

      . use "data_produced/results", clear

      . drop if var=="6.income_group"
      (6 observations deleted)

      . keep if strpos(var , "female")!=0 | strpos(var , "age_large")!=0 | strpos(var , "income_group")!=0
      (118 observations deleted)

      . gen ci_l = coef -1.98*stderr

      . gen ci_r = coef +1.98*stderr

      . replace var = "Male" if var=="0b.female"
      (6 real changes made)

      . replace var = "Female" if var=="1.female"
      (6 real changes made)

      . replace var = "18-25" if var== "1b.age_large"
      (6 real changes made)

      . replace var = "26-45" if var== "2.age_large"
      (6 real changes made)

      . replace var = "46-65" if var== "3.age_large"
      (6 real changes made)

      . replace var = "Above 65" if var== "4.age_large"
      (6 real changes made)

      . replace var = "Income Q1" if var== "1b.income_group"
      (6 real changes made)

      . replace var = "Income Q2" if var== "2.income_group"
      (6 real changes made)

      . replace var = "Income Q3" if var== "3.income_group"
      (6 real changes made)

      . replace var = "Income Q4" if var== "4.income_group"
      (6 real changes made)

      . replace var = "Income Q5" if var== "5.income_group"
      (6 real changes made)

      . sencode var, replace


      . gen asia=1 if inlist(country,"China","Korea","Japan")
      (33 missing values generated)

      . sort asia country

      . sencode country, replace 


      . twoway ///
               (scatter var coef, ///
                       msym(square) mcolor(gs2) by(country, scale(1.8) row(1) ///
                       legend(off) graphregion(color(white) margin(0 0 0 0)) note("")) ///
                       ylabel(1(1)11, glcolor(gs15) val angle(0) labsize(medsmall))) ///
               (rcap ci_l ci_r var, ///
                       color(gs2) horizontal by(country)) ///
               , ytitle("", size(small)) xline(0, lcolor(orange)) ///
               xlabel(, grid glcolor(gs15)) xtitle("") ///
               subtitle(,lcolor(white) fcolor(white)) xsize(9) ysize(3)


      . graph export "figures/age_income_gradients_jobloss_wcontrols_all.png", replace width(850)
      (file figures/age_income_gradients_jobloss_wcontrols_all.png written in PNG format)
```

Figure 1: Age and income gradients on losing job at least temporarily

---

**REPLICATE FIGURE 2**

```
      . use "data_produced/clean_data", clear

      . table country, c(mean started_teleworking) f(%9.3f)

      --------------------------
        country | mean(starte~g)
      ----------+---------------
          China |          0.517
          Japan |          0.188
          Korea |          0.147
          Italy |          0.261
             UK |          0.182
             US |          0.231
      --------------------------

      . qui reg started_teleworking i.age_large i.income i.female ///
               i.current_living_area i.region i.employment_status_pre_pandemic if country==1


      . regsave using "data_produced/results", addlabel(country, China) replace
      file data_produced/results.dta saved

      . forval x=2(1)6 {
      . local country_name : label country `x'
      . qui reg started_teleworking i.age_large i.income i.female ///
               i.current_living_area i.region i.employment_status_pre_pandemic if country==`x'

      . regsave using "data_produced/results", addlabel(country, `country_name') append
      . }
      file data_produced/results.dta saved
      file data_produced/results.dta saved
      file data_produced/results.dta saved
      (note: variable country was str2, now str5 to accommodate using data's values)
      file data_produced/results.dta saved
      (note: variable country was str2, now str5 to accommodate using data's values)
      file data_produced/results.dta saved

      . use "data_produced/results", clear

      . drop if var=="6.income_group"
      (6 observations deleted)

      . keep if strpos(var , "female")!=0 | strpos(var , "age_large")!=0 | strpos(var , "income_group")!=0
      (118 observations deleted)

      . gen ci_l = coef -1.98*stderr

      . gen ci_r = coef +1.98*stderr

      . replace var = "Male" if var=="0b.female"
      (6 real changes made)

      . replace var = "Female" if var=="1.female"
      (6 real changes made)

      . replace var = "18-25" if var== "1b.age_large"
      (6 real changes made)

      . replace var = "26-45" if var== "2.age_large"
      (6 real changes made)

      . replace var = "46-65" if var== "3.age_large"
      (6 real changes made)

      . replace var = "Above 65" if var== "4.age_large"
      (6 real changes made)

      . replace var = "Income Q1" if var== "1b.income_group"
      (6 real changes made)

      . replace var = "Income Q2" if var== "2.income_group"
      (6 real changes made)

      . replace var = "Income Q3" if var== "3.income_group"
      (6 real changes made)

      . replace var = "Income Q4" if var== "4.income_group"
      (6 real changes made)

      . replace var = "Income Q5" if var== "5.income_group"
      (6 real changes made)

      . sencode var, replace


      . gen asia=1 if inlist(country,"China","Korea","Japan")
      (33 missing values generated)

      . sort asia country

      . sencode country, replace 


      . twoway ///
               (scatter var coef, ///
                       msym(square) mcolor(gs2) by(country, scale(1.8) row(1) legend(off) ///
                       graphregion(color(white) margin(0 0 0 0)) note("")) ///
                       ylabel(1(1)11, glcolor(gs15) val angle(0) labsize(medsmall))) ///
               (rcap ci_l ci_r var, color(gs2) horizontal by(country)) ///
               , ytitle("", size(small)) xline(0, lcolor(orange)) ///
               xlabel(-0.8(0.3)0.4, grid glcolor(gs15)) xtitle("") ///
               subtitle(,lcolor(white) fcolor(white)) xsize(9) ysize(3)


      . graph export "figures/age_income_gradients_teleworking_wcontrols_all.png", replace width(850)
      (file figures/age_income_gradients_teleworking_wcontrols_all.png written in PNG format)
```

Figure 2: Age and income gradients on teleworking

---

**REPLICATE FIGURE 3**

```
      . use "data_produced/clean_data", clear

      . table country, c(mean fall_consumption) f(%9.3f)

      --------------------------
        country | mean(fall_c~n)
      ----------+---------------
          China |          0.563
          Japan |          0.308
          Korea |          0.490
          Italy |          0.566
             UK |          0.396
             US |          0.431
      --------------------------

      . qui reg fall_consumption i.age_large i.income i.female ///
               i.current_living_area i.region i.employment_status_pre_pandemic if country==1


      . regsave using "data_produced/results", addlabel(country, China) replace
      file data_produced/results.dta saved

      . forval x=2(1)6 {
      . local country_name : label country `x'
      . qui reg fall_consumption i.age_large i.income i.female ///
               i.current_living_area i.region i.employment_status_pre_pandemic if country==`x'

      . regsave using "data_produced/results", addlabel(country, `country_name') append
      . }
      file data_produced/results.dta saved
      file data_produced/results.dta saved
      file data_produced/results.dta saved
      (note: variable country was str2, now str5 to accommodate using data's values)
      file data_produced/results.dta saved
      (note: variable country was str2, now str5 to accommodate using data's values)
      file data_produced/results.dta saved

      . use "data_produced/results", clear

      . drop if var=="6.income_group"
      (6 observations deleted)

      . keep if strpos(var , "female")!=0 | strpos(var , "age_large")!=0 | strpos(var , "income_group")!=0
      (118 observations deleted)

      . gen ci_l = coef -1.98*stderr

      . gen ci_r = coef +1.98*stderr

      . replace var = "Male" if var=="0b.female"
      (6 real changes made)

      . replace var = "Female" if var=="1.female"
      (6 real changes made)

      . replace var = "18-25" if var== "1b.age_large"
      (6 real changes made)

      . replace var = "26-45" if var== "2.age_large"
      (6 real changes made)

      . replace var = "46-65" if var== "3.age_large"
      (6 real changes made)

      . replace var = "Above 65" if var== "4.age_large"
      (6 real changes made)

      . replace var = "Income Q1" if var== "1b.income_group"
      (6 real changes made)

      . replace var = "Income Q2" if var== "2.income_group"
      (6 real changes made)

      . replace var = "Income Q3" if var== "3.income_group"
      (6 real changes made)

      . replace var = "Income Q4" if var== "4.income_group"
      (6 real changes made)

      . replace var = "Income Q5" if var== "5.income_group"
      (6 real changes made)

      . sencode var, replace


      . gen asia=1 if inlist(country,"China","Korea","Japan")
      (33 missing values generated)

      . sort asia country

      . sencode country, replace 


      . twoway ///
               (scatter var coef, ///
                       msym(square) mcolor(gs2) by(country, scale(1.8) row(1) legend(off) ///
                       graphregion(color(white) margin(0 0 0 0))  note("")) ///
                       ylabel(1(1)11, glcolor(gs15) val angle(0) labsize(medsmall))) ///
               (rcap ci_l ci_r var, ///
                       color(gs2) horizontal by(country)) ///
               , ytitle("", size(small)) xline(0, lcolor(orange)) ///
               xlabel(, grid glcolor(gs15)) xtitle("") ///
               subtitle(,lcolor(white) fcolor(white)) xsize(9) ysize(3)


      . graph export "figures/age_income_gradients_consumptiondrop_wcontrols_all.png", replace width(850)
      (file figures/age_income_gradients_consumptiondrop_wcontrols_all.png written in PNG format)
```

Figure 3: Age and income gradients on drop in household spending

---

**REPLICATE FIGURE 4**

```
      . use "data_produced/clean_data", clear

      . egen avg_interactions_1=rowmean(Q291_8 Q291_9 Q291_10 /*Q291_11 Q291_14*/ Q291_12   )

      . egen avg_interactions_2=rowmean(Q292_8 Q292_9 Q292_10 /*Q292_11 Q292_14*/ Q292_12  )

      . egen avg_interactions_3=rowmean(Q293_8 Q293_9 Q293_10 /*Q293_11 Q293_14*/ Q293_12  )

      . preserve

      . collapse (mean) avg_interactions_1 avg_interactions_2 avg_interactions_3, by(country age_large)

      . keep if age_large!=.
      (3 observations deleted)

      . label define age_large_lab 1 "18-25" 2 "26-45" 3 "46-65" 4 "Above 66"

      . label values age_large age_large_lab

      . rename age_large group


      . mkmat * ,mat(A)


      . restore

      . preserve

      . collapse (mean) avg_interactions_1 avg_interactions_2 avg_interactions_3, by(country income_group)

      . keep if income_group<6
      (6 observations deleted)

      . rename income_group group


      . replace group = group+4
      (30 real changes made)

      . mkmat * ,mat(B)


      . restore

      . mat C=A\B

      . clear

      . svmat C
      number of observations will be reset to 54
      Press any key to continue, or Break to abort
      number of observations (_N) was 0, now 54

      . rename C1 country


      . rename C2 group


      . la def country 1 "China" 2 "Japan" 3 "Korea" 4 "Italy" 5 "UK" 6 "US" 

      . la val country country

      . label define group 1 "18-25" 2 "26-45" 3 "46-65" 4 "Above 66" 5 "Income Q1" 6 "Income Q2" 7 "Income Q3" 8 "Income Q4" 9 "Income Q5"

      . la val group group

      . twoway ///
               (scatter group C3, ///
                       by(country, row(1) scale(1.8)  note("") graphregion(color(white) ///
                       margin(0 0 0 0))) msym(X) msize(medlarge) ///
                       ylabel(1(1)9, glcolor(gs15) val angle(0)  )) ///
               (scatter group C4, ///
                       by(country) msym(X) msize(medlarge)) ///
               (scatter group C5, ///
                       by(country) msym(X) msize(medlarge)) ///+
               , subtitle(,lcolor(white) fcolor(white)) ///
               legend(order(1 "Normal times" 2 "Start pandemic" 3 "Time of survey") row(1) ///
               size(small) bmargin(zero) symxsize(5) region(lcolor(white))) ytitle("") ///
               xscale(range(1 5))  xlabel(1 "Never" 2 "Rarely" 3 "Sometimes" ///
               4 "Very often" 5 "Always" , grid glcolor(gs15) angle(35) labsize(small)) ///
               xsize(9) ysize(3) name(gr2, replace)


      . graph export "figures/age_income_means_social_interactions.png", replace width(850)
      (file figures/age_income_means_social_interactions.png written in PNG format)
```

Figure 4: Social interactions over time, by age and income groups

---

**REPLICATE FIGURE 5**

```
      . use "data_produced/clean_data", clear

      . table country, c(mean neg_non_fin) f(%9.3f)

      --------------------------
        country | mean(neg_no~n)
      ----------+---------------
          China |          1.673
          Japan |          1.229
          Korea |          1.410
          Italy |          1.640
             UK |          1.659
             US |          1.693
      --------------------------

      . qui reg neg_non_fin i.age_large i.income i.female i.current_living_area i.region i.employment_status_pre_pandemic if country==1

      . regsave using "data_produced/results", addlabel(country, China) replace
      file data_produced/results.dta saved

      . forval x=2(1)6 {
      . local country_name : label country `x'
      . qui reg neg_non_fin i.age_large i.income i.female i.current_living_area i.region i.employment_status_pre_pandemic if country==`x'
      . regsave using "data_produced/results", addlabel(country, `country_name') append
      . }
      file data_produced/results.dta saved
      file data_produced/results.dta saved
      file data_produced/results.dta saved
      (note: variable country was str2, now str5 to accommodate using data's values)
      file data_produced/results.dta saved
      (note: variable country was str2, now str5 to accommodate using data's values)
      file data_produced/results.dta saved

      . use "data_produced/results", clear

      . drop if var=="6.income_group"
      (6 observations deleted)

      . keep if strpos(var , "female")!=0 | strpos(var , "age_large")!=0 | strpos(var , "income_group")!=0
      (118 observations deleted)

      . gen ci_l = coef -1.98*stderr

      . gen ci_r = coef +1.98*stderr

      . replace var = "Male" if var=="0b.female"
      (6 real changes made)

      . replace var = "Female" if var=="1.female"
      (6 real changes made)

      . replace var = "18-25" if var== "1b.age_large"
      (6 real changes made)

      . replace var = "26-45" if var== "2.age_large"
      (6 real changes made)

      . replace var = "46-65" if var== "3.age_large"
      (6 real changes made)

      . replace var = "Above 65" if var== "4.age_large"
      (6 real changes made)

      . replace var = "Income Q1" if var== "1b.income_group"
      (6 real changes made)

      . replace var = "Income Q2" if var== "2.income_group"
      (6 real changes made)

      . replace var = "Income Q3" if var== "3.income_group"
      (6 real changes made)

      . replace var = "Income Q4" if var== "4.income_group"
      (6 real changes made)

      . replace var = "Income Q5" if var== "5.income_group"
      (6 real changes made)

      . sencode var, replace


      . gen asia=1 if inlist(country,"China","Korea","Japan")
      (33 missing values generated)

      . sort asia country

      . sencode country, replace 


      . twoway ///
               (scatter var coef, ///
                       msym(square) mcolor(gs2) by(country, scale(1.8) row(1) legend(off) ///
                       graphregion(color(white) margin(0 0 0 0))  note("")) ///
               ylabel(1(1)11, glcolor(gs15) val angle(0) labsize(medsmall))) ///
               (rcap ci_l ci_r var, ///
                       color(gs2) horizontal by(country)) ///
               , ytitle("", size(small)) xline(0, lcolor(orange)) ///
               xlabel(, grid glcolor(gs15)) xtitle("") ///
               subtitle(,lcolor(white) fcolor(white)) xsize(9) ysize(3)


      . graph export "figures/age_income_gradients_neg_non_fin_wcontrols_all.png", replace width(850)
      (file figures/age_income_gradients_neg_non_fin_wcontrols_all.png written in PNG format)
```

Figure 5: Age and income gradients on negative well-being

---

**REPLICATE FIGURE 6**

```
      . use "data_produced/clean_data", clear

      . collapse (mean) neg_nonfin_anxiety neg_nonfin_boredom neg_nonfin_conflicts neg_nonfin_loneliness neg_nonfin_troublesleep, by(country age_large)

      . drop if age_large==.
      (3 observations deleted)

      . twoway ///
               (scatter neg_nonfin_anxiety age_large, ///
                       by(country, graphregion(color(white) margin(0 0 0 0))  note("")) ///
                       mcolor(emerald) msym(o)) ///
               (scatter neg_nonfin_boredom age_large, ///
                       by(country) mcolor(gold) msym(d)) ///
               (scatter neg_nonfin_conflicts age_large, ///
                       by(country) mcolor(cranberry) msym(t)) ///
               (scatter neg_nonfin_loneliness age_large, ///
                       by(country) mcolor(forest_green) msym(s)) ///
               (scatter neg_nonfin_troublesleep age_large, ///
                       by(country) mcolor(lavender) msize(large) msym(smplus)) ///
               (lfit neg_nonfin_anxiety age_large, ///
                       by(country) lcolor(emerald) lp(.-.)) ///
               (lfit neg_nonfin_boredom age_large, ///
                       by(country) lcolor(gold) lp(.-.)) ///
               (lfit neg_nonfin_conflicts age_large, ///
                       by(country) lcolor(cranberry) lp(.-.)) ///
               (lfit neg_nonfin_loneliness age_large, ///
                       by(country) lcolor(forest_green) lp(.-.)) ///
               (lfit neg_nonfin_troublesleep age_large, ///
                       by(country) lcolor(lavender) lp(.-.)) ///
               , xtitle("Age group") ///
               xlabel(1 "18-25" 2 "26-45" 3 "46-65" 4 "66+", grid glcolor(gs15)) ///
               ytitle("Percentage reporting non-financial distress") ///
               legend(order(1 "Anxiety" 2 "Boredom" 3 "Conflicts" 4 "Loneliness" 5 "Trouble sleeping") ///
               row(1) size(small) bmargin(zero) symxsize(5) region(lcolor(white))) ///
               xsize(9) ysize(6) scale(1.8) subtitle(,lcolor(white) fcolor(white))


      . graph export "figures/age_gradients_nonfin.png", replace width(850)
      (file figures/age_gradients_nonfin.png written in PNG format)
```

Figure 6: Age gradients on negative well-being

---

```
      . use "data_produced/clean_data", clear

      . lab def age_large 1 "Age below 26" 2 "Age 26-45" 3 "Age 46-65" 4 "Age above 66"

      . lab val age_large age_large

      . lab var female "Female"

      . lab var recent_cases_percapita "Recent cases per capita"
```

**REPLICATE TABLE A1**

```
      . qui eststo country1: reg lost_job_atleast_temporarily i.age_large i.income female i.region i.current_living_area i.employment_status_pre_pandemic if country==1

      . qui eststo country2: reg lost_job_atleast_temporarily i.age_large i.income female i.region i.current_living_area i.employment_status_pre_pandemic if country==2

      . qui eststo country3: reg lost_job_atleast_temporarily i.age_large i.income female i.region i.current_living_area i.employment_status_pre_pandemic if country==3

      . qui eststo country4: reg lost_job_atleast_temporarily i.age_large i.income female i.region i.current_living_area i.employment_status_pre_pandemic if country==4

      . qui eststo country5: reg lost_job_atleast_temporarily i.age_large i.income female i.region i.current_living_area i.employment_status_pre_pandemic if country==5

      . qui eststo country6: reg lost_job_atleast_temporarily i.age_large i.income female i.region i.current_living_area i.employment_status_pre_pandemic if country==6

      . qui eststo all: reg lost_job_atleast_temporarily i.age_large i.income female i.country i.region i.current_living_area i.employment_status_pre_pandemic

      . qui eststo all2: reg lost_job_atleast_temporarily i.age_large i.income female i.country i.current_living_area i.employment_status_pre_pandemic c.recent_cases
```

Table A1: Linear probability model for having lost the job at least temporarily

```
      . estout country1 country2 country3 country4 country5 country6 all all2, ///
               ce(b(star fmt(3) label("Coef")) se(par fmt(3))) nobaselevels label ///
               varwidth(35) stats(N r2_a, fmt(0 3)) title("TABLE A1") ///
               starl(* 0.10 ** 0.05 *** 0.01) ///
               keep(*.income_group *.age_large female *.current_living_area *.employment_status_pre_pandemic recent_cases_percapita _cons) ///
               drop(6.income_group) mlabels("China" "Japan" "Korea" "Italy" "UK"  "US") ///
               indicate("Regional fixed effects = *region" "Country fixed effects = *country") ///
               order(female *age_large *income_group *current_living_area *employment_status_pre_pandemic recent_cases_percapita)


      TABLE A1
      -------------------------------------------------------------------------------------------------------------------------------------------------------------------
                                                 China           Japan           Korea           Italy              UK              US             all            all2   
                                               Coef/se         Coef/se         Coef/se         Coef/se         Coef/se         Coef/se         Coef/se         Coef/se   
      -------------------------------------------------------------------------------------------------------------------------------------------------------------------
      Female                                    -0.029           0.055**         0.079***        0.033          -0.010           0.037           0.026**         0.027***
                                               (0.030)         (0.022)         (0.026)         (0.026)         (0.026)         (0.026)         (0.011)         (0.011)   
      Age 26-45                                 -0.000          -0.135***       -0.045          -0.043          -0.068          -0.052          -0.052***       -0.052***
                                               (0.046)         (0.039)         (0.042)         (0.045)         (0.044)         (0.043)         (0.018)         (0.017)   
      Age 46-65                                  0.074          -0.130***       -0.047          -0.100**        -0.130***       -0.112***       -0.069***       -0.065***
                                               (0.051)         (0.038)         (0.042)         (0.045)         (0.044)         (0.043)         (0.018)         (0.017)   
      Age above 66                               0.183***       -0.061          -0.005          -0.014          -0.104*         -0.080          -0.011          -0.006   
                                               (0.066)         (0.045)         (0.049)         (0.054)         (0.054)         (0.049)         (0.021)         (0.021)   
      Second quintile                           -0.032          -0.054          -0.137***       -0.064          -0.025          -0.024          -0.078***       -0.078***
                                               (0.049)         (0.035)         (0.042)         (0.044)         (0.043)         (0.041)         (0.017)         (0.017)   
      Third quintile                            -0.094*         -0.105***       -0.181***       -0.167***        0.019          -0.096**        -0.131***       -0.130***
                                               (0.050)         (0.035)         (0.040)         (0.043)         (0.042)         (0.040)         (0.017)         (0.017)   
      Fourth quintile                           -0.189***       -0.104***       -0.237***       -0.182***       -0.169***       -0.095**        -0.191***       -0.191***
                                               (0.050)         (0.037)         (0.040)         (0.042)         (0.041)         (0.040)         (0.017)         (0.017)   
      Fifth quintile                            -0.260***       -0.110***       -0.264***       -0.274***       -0.182***       -0.163***       -0.236***       -0.237***
                                               (0.054)         (0.039)         (0.043)         (0.047)         (0.043)         (0.043)         (0.018)         (0.018)   
      Semi-urban / residential                   0.121***       -0.073***        0.011          -0.014           0.001          -0.031           0.005          -0.001   
                                               (0.039)         (0.026)         (0.037)         (0.031)         (0.029)         (0.028)         (0.013)         (0.012)   
      Countryside                                0.133**        -0.050*         -0.103          -0.028           0.075*         -0.086**        -0.013          -0.022   
                                               (0.060)         (0.030)         (0.064)         (0.038)         (0.042)         (0.044)         (0.017)         (0.017)   
      Employed before pandemic                   0.642***        0.255***        0.437***        0.578***        0.442***        0.518***        0.477***        0.479***
                                               (0.066)         (0.030)         (0.030)         (0.034)         (0.033)         (0.032)         (0.014)         (0.014)   
      Recent cases per capita                                                                                                                                    3.852   
                                                                                                                                                               (2.959)   
      _cons                                     -0.169*          0.159***        0.172***        0.118           0.073           0.117**         0.107*          0.145***
                                               (0.102)         (0.057)         (0.061)         (0.104)         (0.070)         (0.059)         (0.059)         (0.025)   
      Regional fixed effects                       Yes             Yes             Yes             Yes             Yes             Yes             Yes              No   
      Country fixed effects                         No              No              No              No              No              No             Yes             Yes   
      -------------------------------------------------------------------------------------------------------------------------------------------------------------------
      N                                            996            1013             962            1042            1016            1055            6084            6084   
      r2_a                                       0.141           0.095           0.210           0.292           0.232           0.292           0.229           0.228   
      -------------------------------------------------------------------------------------------------------------------------------------------------------------------
```

---

**REPLICATE TABLE A2**

```
      . qui eststo country1: reg started_teleworking i.age_large i.income female i.region i.current_living_area i.employment_status_pre_pandemic if country==1

      . qui eststo country2: reg started_teleworking i.age_large i.income female i.region i.current_living_area i.employment_status_pre_pandemic if country==2

      . qui eststo country3: reg started_teleworking i.age_large i.income female i.region i.current_living_area i.employment_status_pre_pandemic if country==3

      . qui eststo country4: reg started_teleworking i.age_large i.income female i.region i.current_living_area i.employment_status_pre_pandemic if country==4

      . qui eststo country5: reg started_teleworking i.age_large i.income female i.region i.current_living_area i.employment_status_pre_pandemic if country==5

      . qui eststo country6: reg started_teleworking i.age_large i.income female i.region i.current_living_area i.employment_status_pre_pandemic if country==6

      . qui eststo all: reg started_teleworking i.age_large i.income female i.country i.region i.current_living_area i.employment_status_pre_pandemic

      . qui eststo all2: reg started_teleworking i.age_large i.income female i.country i.current_living_area i.employment_status_pre_pandemic c.recent_cases
```

Table A2: Linear probability model for having started teleworking

```
      . estout country1 country2 country3 country4 country5 country6 all all2, ///
               ce(b(star fmt(3) label("Coef")) se(par fmt(3))) nobaselevels label ///
               varwidth(35) stats(N r2_a, fmt(0 3)) title("TABLE A2") ///
               starl(* 0.10 ** 0.05 *** 0.01) ///
               keep(*.income_group *.age_large female *.current_living_area *.employment_status_pre_pandemic recent_cases_percapita _cons) ///
               drop(6.income_group) mlabels("China" "Japan" "Korea" "Italy" "UK"  "US") ///
               indicate("Regional fixed effects = *region" "Country fixed effects = *country") ///
               order(female *age_large *income_group *current_living_area *employment_status_pre_pandemic recent_cases_percapita)


      TABLE A2
      -------------------------------------------------------------------------------------------------------------------------------------------------------------------
                                                 China           Japan           Korea           Italy              UK              US             all            all2   
                                               Coef/se         Coef/se         Coef/se         Coef/se         Coef/se         Coef/se         Coef/se         Coef/se   
      -------------------------------------------------------------------------------------------------------------------------------------------------------------------
      Female                                    -0.004          -0.030          -0.027          -0.008          -0.086***       -0.065***       -0.041***       -0.041***
                                               (0.029)         (0.023)         (0.023)         (0.025)         (0.023)         (0.024)         (0.010)         (0.010)   
      Age 26-45                                 -0.117***       -0.046          -0.097***       -0.020           0.006           0.148***       -0.018          -0.023   
                                               (0.044)         (0.041)         (0.037)         (0.044)         (0.039)         (0.039)         (0.017)         (0.017)   
      Age 46-65                                 -0.252***       -0.090**        -0.156***       -0.030          -0.069*          0.031          -0.093***       -0.101***
                                               (0.048)         (0.041)         (0.038)         (0.044)         (0.040)         (0.039)         (0.017)         (0.017)   
      Age above 66                              -0.523***       -0.148***       -0.127***       -0.083          -0.084*         -0.024          -0.163***       -0.167***
                                               (0.063)         (0.048)         (0.044)         (0.053)         (0.049)         (0.044)         (0.020)         (0.020)   
      Second quintile                            0.213***        0.030           0.007           0.056          -0.012           0.061           0.069***        0.076***
                                               (0.047)         (0.038)         (0.037)         (0.043)         (0.039)         (0.037)         (0.016)         (0.016)   
      Third quintile                             0.067           0.097***        0.125***        0.129***        0.049           0.125***        0.114***        0.111***
                                               (0.048)         (0.037)         (0.036)         (0.042)         (0.038)         (0.036)         (0.016)         (0.016)   
      Fourth quintile                            0.113**         0.148***        0.107***        0.185***        0.100***        0.223***        0.164***        0.170***
                                               (0.048)         (0.040)         (0.036)         (0.041)         (0.037)         (0.036)         (0.016)         (0.016)   
      Fifth quintile                             0.165***        0.267***        0.133***        0.297***        0.161***        0.266***        0.241***        0.252***
                                               (0.052)         (0.041)         (0.038)         (0.046)         (0.038)         (0.039)         (0.017)         (0.017)   
      Semi-urban / residential                  -0.130***       -0.004          -0.032          -0.083***       -0.034          -0.035          -0.069***       -0.067***
                                               (0.037)         (0.028)         (0.033)         (0.030)         (0.027)         (0.025)         (0.012)         (0.011)   
      Countryside                               -0.241***       -0.023          -0.028          -0.103***       -0.054          -0.105***       -0.100***       -0.103***
                                               (0.058)         (0.032)         (0.058)         (0.037)         (0.038)         (0.040)         (0.017)         (0.016)   
      Employed before pandemic                   0.150**         0.151***        0.129***        0.269***        0.177***        0.187***        0.165***        0.169***
                                               (0.063)         (0.032)         (0.027)         (0.033)         (0.030)         (0.029)         (0.013)         (0.013)   
      Recent cases per capita                                                                                                                                   12.500***
                                                                                                                                                               (2.821)   
      _cons                                      0.533***        0.038           0.107*          0.060           0.043          -0.032           0.357***        0.342***
                                               (0.098)         (0.061)         (0.055)         (0.102)         (0.063)         (0.053)         (0.056)         (0.024)   
      Regional fixed effects                       Yes             Yes             Yes             Yes             Yes             Yes             Yes              No   
      Country fixed effects                         No              No              No              No              No              No             Yes             Yes   
      -------------------------------------------------------------------------------------------------------------------------------------------------------------------
      N                                            996            1013             962            1042            1016            1055            6084            6084   
      r2_a                                       0.231           0.166           0.058           0.178           0.139           0.286           0.231           0.222   
      -------------------------------------------------------------------------------------------------------------------------------------------------------------------
```

---

**REPLICATE TABLE A3**

```
      . qui eststo country1: reg fall_consumption i.age_large i.income female i.region i.current_living_area i.employment_status_pre_pandemic if country==1

      . qui eststo country2: reg fall_consumption i.age_large i.income female i.region i.current_living_area i.employment_status_pre_pandemic if country==2

      . qui eststo country3: reg fall_consumption i.age_large i.income female i.region i.current_living_area i.employment_status_pre_pandemic if country==3

      . qui eststo country4: reg fall_consumption i.age_large i.income female i.region i.current_living_area i.employment_status_pre_pandemic if country==4

      . qui eststo country5: reg fall_consumption i.age_large i.income female i.region i.current_living_area i.employment_status_pre_pandemic if country==5

      . qui eststo country6: reg fall_consumption i.age_large i.income female i.region i.current_living_area i.employment_status_pre_pandemic if country==6

      . qui eststo all: reg fall_consumption i.age_large i.income female i.country i.region i.current_living_area i.employment_status_pre_pandemic

      . qui eststo all2: reg fall_consumption i.age_large i.income female i.country i.current_living_area i.employment_status_pre_pandemic c.recent_cases
```

Table A3: Linear probability model for having experienced drop in spending

```
      . estout country1 country2 country3 country4 country5 country6 all all2, ///
               ce(b(star fmt(3) label("Coef")) se(par fmt(3))) nobaselevels label ///
               varwidth(35) stats(N r2_a, fmt(0 3)) title("TABLE A3") ///
               starl(* 0.10 ** 0.05 *** 0.01) ///
               keep(*.income_group *.age_large female *.current_living_area *.employment_status_pre_pandemic recent_cases_percapita _cons) ///
               drop(6.income_group) mlabels("China" "Japan" "Korea" "Italy" "UK"  "US") ///
               indicate("Regional fixed effects = *region" "Country fixed effects = *country") ///
               order(female *age_large *income_group *current_living_area *employment_status_pre_pandemic recent_cases_percapita)


      TABLE A3
      -------------------------------------------------------------------------------------------------------------------------------------------------------------------
                                                 China           Japan           Korea           Italy              UK              US             all            all2   
                                               Coef/se         Coef/se         Coef/se         Coef/se         Coef/se         Coef/se         Coef/se         Coef/se   
      -------------------------------------------------------------------------------------------------------------------------------------------------------------------
      Female                                     0.035           0.051*          0.050           0.013          -0.078***       -0.045          -0.002          -0.000   
                                               (0.032)         (0.030)         (0.034)         (0.031)         (0.030)         (0.031)         (0.013)         (0.013)   
      Age 26-45                                 -0.053          -0.092*         -0.070           0.079          -0.012           0.033          -0.028          -0.029   
                                               (0.049)         (0.053)         (0.054)         (0.054)         (0.051)         (0.052)         (0.021)         (0.021)   
      Age 46-65                                 -0.119**        -0.172***       -0.013           0.064          -0.006          -0.028          -0.056***       -0.060***
                                               (0.054)         (0.052)         (0.054)         (0.054)         (0.052)         (0.051)         (0.021)         (0.021)   
      Age above 66                              -0.265***       -0.133**        -0.033           0.035           0.002          -0.019          -0.070***       -0.072***
                                               (0.070)         (0.062)         (0.063)         (0.065)         (0.064)         (0.059)         (0.025)         (0.025)   
      Second quintile                            0.080          -0.027          -0.058           0.029           0.073           0.097**         0.033           0.032   
                                               (0.052)         (0.048)         (0.053)         (0.053)         (0.051)         (0.049)         (0.021)         (0.020)   
      Third quintile                             0.025          -0.017          -0.111**        -0.030           0.192***        0.098**         0.031           0.030   
                                               (0.053)         (0.048)         (0.051)         (0.051)         (0.050)         (0.048)         (0.020)         (0.020)   
      Fourth quintile                           -0.017          -0.017          -0.034           0.021           0.090*          0.128***        0.040**         0.038*  
                                               (0.054)         (0.051)         (0.051)         (0.051)         (0.049)         (0.048)         (0.020)         (0.020)   
      Fifth quintile                            -0.092          -0.029          -0.083          -0.052           0.116**         0.189***        0.026           0.030   
                                               (0.058)         (0.053)         (0.055)         (0.057)         (0.050)         (0.052)         (0.021)         (0.021)   
      Semi-urban / residential                   0.010           0.008          -0.081*         -0.017          -0.037           0.038          -0.017          -0.019   
                                               (0.042)         (0.036)         (0.048)         (0.037)         (0.034)         (0.034)         (0.015)         (0.014)   
      Countryside                               -0.011          -0.028          -0.102          -0.077*          0.020          -0.014          -0.035*         -0.042** 
                                               (0.065)         (0.041)         (0.082)         (0.045)         (0.050)         (0.053)         (0.021)         (0.020)   
      Employed before pandemic                   0.069           0.114***        0.110***        0.103**         0.212***        0.207***        0.145***        0.148***
                                               (0.071)         (0.042)         (0.038)         (0.041)         (0.039)         (0.039)         (0.017)         (0.017)   
      Recent cases per capita                                                                                                                                    5.022   
                                                                                                                                                               (3.547)   
      _cons                                      0.455***        0.296***        0.453***        0.365***        0.181**         0.253***        0.323***        0.446***
                                               (0.109)         (0.078)         (0.079)         (0.126)         (0.082)         (0.071)         (0.070)         (0.030)   
      Regional fixed effects                       Yes             Yes             Yes             Yes             Yes             Yes             Yes              No   
      Country fixed effects                         No              No              No              No              No              No             Yes             Yes   
      -------------------------------------------------------------------------------------------------------------------------------------------------------------------
      N                                            996            1013             962            1042            1016            1055            6084            6084   
      r2_a                                       0.026           0.019           0.026           0.022           0.094           0.086           0.066           0.059   
      -------------------------------------------------------------------------------------------------------------------------------------------------------------------
```

---

**REPLICATE TABLE A4**

```
      . qui eststo country1: reg neg_non_fin i.age_large i.income female i.region i.current_living_area i.employment_status_pre_pandemic if country==1

      . qui eststo country2: reg neg_non_fin i.age_large i.income female i.region i.current_living_area i.employment_status_pre_pandemic if country==2

      . qui eststo country3: reg neg_non_fin i.age_large i.income female i.region i.current_living_area i.employment_status_pre_pandemic if country==3

      . qui eststo country4: reg neg_non_fin i.age_large i.income female i.region i.current_living_area i.employment_status_pre_pandemic if country==4

      . qui eststo country5: reg neg_non_fin i.age_large i.income female i.region i.current_living_area i.employment_status_pre_pandemic if country==5

      . qui eststo country6: reg neg_non_fin i.age_large i.income female i.region i.current_living_area i.employment_status_pre_pandemic if country==6

      . qui eststo all: reg neg_non_fin i.age_large i.income female i.country i.region i.current_living_area i.employment_status_pre_pandemic 

      . qui eststo all2: reg neg_non_fin i.age_large i.income female i.country i.current_living_area i.employment_status_pre_pandemic c.recent_cases
```

Table A4: Ordinary least square for negative well-being

```
      . estout country1 country2 country3 country4 country5 country6 all all2, ///
               ce(b(star fmt(3) label("Coef")) se(par fmt(3))) nobaselevels label ///
               varwidth(35) stats(N r2_a, fmt(0 3)) title("TABLE A4") ///
               starl(* 0.10 ** 0.05 *** 0.01) ///
               keep(*.income_group *.age_large female *.current_living_area *.employment_status_pre_pandemic recent_cases_percapita _cons) ///
               drop(6.income_group) mlabels("China" "Japan" "Korea" "Italy" "UK"  "US") ///
               indicate("Regional fixed effects = *region" "Country fixed effects = *country") ///
               order(female *age_large *income_group *current_living_area *employment_status_pre_pandemic recent_cases_percapita)


      TABLE A4
      -------------------------------------------------------------------------------------------------------------------------------------------------------------------
                                                 China           Japan           Korea           Italy              UK              US             all            all2   
                                               Coef/se         Coef/se         Coef/se         Coef/se         Coef/se         Coef/se         Coef/se         Coef/se   
      -------------------------------------------------------------------------------------------------------------------------------------------------------------------
      Female                                     0.028           0.400***        0.257***        0.292***        0.405***        0.416***        0.300***        0.299***
                                               (0.067)         (0.068)         (0.072)         (0.074)         (0.085)         (0.086)         (0.031)         (0.030)   
      Age 26-45                                 -0.326***       -0.315***       -0.195*         -0.403***       -0.328**        -0.228          -0.311***       -0.312***
                                               (0.101)         (0.120)         (0.116)         (0.128)         (0.145)         (0.141)         (0.051)         (0.050)   
      Age 46-65                                 -0.583***       -0.558***       -0.399***       -0.581***       -0.566***       -0.766***       -0.571***       -0.574***
                                               (0.111)         (0.119)         (0.118)         (0.129)         (0.147)         (0.140)         (0.051)         (0.051)   
      Age above 66                              -0.430***       -0.588***       -0.472***       -0.810***       -1.084***       -1.034***       -0.773***       -0.782***
                                               (0.145)         (0.140)         (0.135)         (0.154)         (0.181)         (0.160)         (0.060)         (0.060)   
      Second quintile                            0.010           0.007          -0.075           0.137          -0.173           0.105          -0.012          -0.012   
                                               (0.108)         (0.109)         (0.115)         (0.127)         (0.143)         (0.134)         (0.050)         (0.049)   
      Third quintile                             0.120          -0.093           0.066          -0.045          -0.209           0.062          -0.043          -0.057   
                                               (0.109)         (0.109)         (0.110)         (0.122)         (0.141)         (0.132)         (0.049)         (0.048)   
      Fourth quintile                           -0.043           0.042          -0.084          -0.025          -0.390***        0.104          -0.079          -0.086*  
                                               (0.111)         (0.115)         (0.111)         (0.121)         (0.138)         (0.130)         (0.049)         (0.049)   
      Fifth quintile                             0.085           0.069          -0.055          -0.089          -0.318**        -0.082          -0.080          -0.074   
                                               (0.119)         (0.120)         (0.119)         (0.135)         (0.141)         (0.142)         (0.052)         (0.051)   
      Semi-urban / residential                   0.287***       -0.034          -0.050           0.037           0.107           0.086           0.072**         0.054   
                                               (0.086)         (0.082)         (0.104)         (0.088)         (0.098)         (0.091)         (0.037)         (0.034)   
      Countryside                                0.034          -0.025          -0.328*         -0.359***       -0.052          -0.041          -0.122**        -0.162***
                                               (0.133)         (0.093)         (0.178)         (0.108)         (0.141)         (0.144)         (0.050)         (0.048)   
      Employed before pandemic                   0.856***        0.363***        0.079           0.144           0.200*          0.160           0.243***        0.240***
                                               (0.145)         (0.095)         (0.082)         (0.098)         (0.110)         (0.106)         (0.041)         (0.040)   
      Recent cases per capita                                                                                                                                   15.178*  
                                                                                                                                                               (8.566)   
      _cons                                      0.809***        1.188***        1.632***        1.724***        1.767***        1.820***        1.403***        1.745***
                                               (0.225)         (0.178)         (0.170)         (0.301)         (0.231)         (0.193)         (0.170)         (0.073)   
      Regional fixed effects                       Yes             Yes             Yes             Yes             Yes             Yes             Yes              No   
      Country fixed effects                         No              No              No              No              No              No             Yes             Yes   
      -------------------------------------------------------------------------------------------------------------------------------------------------------------------
      N                                            996            1013             962            1042            1016            1055            6084            6084   
      r2_a                                       0.116           0.096           0.032           0.088           0.090           0.106           0.098           0.092   
      -------------------------------------------------------------------------------------------------------------------------------------------------------------------
```

---

**REPLICATE TABLE A5**

```
      . qui eststo country1: reg pos_non_fin i.age_large i.income female i.region i.current_living_area i.employment_status_pre_pandemic if country==1

      . qui eststo country2: reg pos_non_fin i.age_large i.income female i.region i.current_living_area i.employment_status_pre_pandemic if country==2

      . qui eststo country3: reg pos_non_fin i.age_large i.income female i.region i.current_living_area i.employment_status_pre_pandemic if country==3

      . qui eststo country4: reg pos_non_fin i.age_large i.income female i.region i.current_living_area i.employment_status_pre_pandemic if country==4

      . qui eststo country5: reg pos_non_fin i.age_large i.income female i.region i.current_living_area i.employment_status_pre_pandemic if country==5

      . qui eststo country6: reg pos_non_fin i.age_large i.income female i.region i.current_living_area i.employment_status_pre_pandemic if country==6

      . qui eststo all: reg pos_non_fin i.age_large i.income female i.country i.region i.current_living_area i.employment_status_pre_pandemic

      . qui eststo all2: reg pos_non_fin i.age_large i.income female i.country i.current_living_area i.employment_status_pre_pandemic c.recent_cases
```

Table A5: Ordinary least square for positive well-being

```
      . estout country1 country2 country3 country4 country5 country6 all all2, ///
               ce(b(star fmt(3) label("Coef")) se(par fmt(3))) nobaselevels label ///
               varwidth(35) stats(N r2_a, fmt(0 3)) title("TABLE A5") ///
               starl(* 0.10 ** 0.05 *** 0.01) ///
               keep(*.income_group *.age_large female *.current_living_area *.employment_status_pre_pandemic recent_cases_percapita _cons) ///
               drop(6.income_group) mlabels("China" "Japan" "Korea" "Italy" "UK"  "US") ///
               indicate("Regional fixed effects = *region" "Country fixed effects = *country") ///
               order(female *age_large *income_group *current_living_area *employment_status_pre_pandemic recent_cases_percapita)


      TABLE A5
      -------------------------------------------------------------------------------------------------------------------------------------------------------------------
                                                 China           Japan           Korea           Italy              UK              US             all            all2   
                                               Coef/se         Coef/se         Coef/se         Coef/se         Coef/se         Coef/se         Coef/se         Coef/se   
      -------------------------------------------------------------------------------------------------------------------------------------------------------------------
      Female                                     0.010           0.002           0.093          -0.009           0.103           0.095           0.037           0.037   
                                               (0.062)         (0.051)         (0.060)         (0.063)         (0.069)         (0.069)         (0.025)         (0.025)   
      Age 26-45                                 -0.096          -0.147*         -0.174*          0.003          -0.242**         0.055          -0.118***       -0.121***
                                               (0.095)         (0.089)         (0.095)         (0.109)         (0.118)         (0.114)         (0.042)         (0.042)   
      Age 46-65                                 -0.453***       -0.124          -0.269***        0.093           0.053          -0.073          -0.132***       -0.137***
                                               (0.103)         (0.089)         (0.097)         (0.110)         (0.119)         (0.113)         (0.042)         (0.042)   
      Age above 66                              -0.447***        0.069          -0.367***        0.122           0.292**        -0.140          -0.112**        -0.109** 
                                               (0.135)         (0.104)         (0.111)         (0.131)         (0.146)         (0.129)         (0.050)         (0.050)   
      Second quintile                            0.025           0.067           0.224**         0.132          -0.201*          0.134           0.085**         0.087** 
                                               (0.100)         (0.081)         (0.095)         (0.108)         (0.116)         (0.108)         (0.041)         (0.041)   
      Third quintile                             0.063           0.096           0.152*          0.227**        -0.192*          0.224**         0.127***        0.120***
                                               (0.102)         (0.081)         (0.091)         (0.104)         (0.114)         (0.106)         (0.040)         (0.040)   
      Fourth quintile                           -0.082           0.077           0.219**         0.145          -0.060           0.109           0.112***        0.115***
                                               (0.103)         (0.086)         (0.092)         (0.103)         (0.112)         (0.105)         (0.041)         (0.040)   
      Fifth quintile                             0.079           0.127           0.040           0.256**        -0.084           0.060           0.135***        0.150***
                                               (0.111)         (0.089)         (0.098)         (0.115)         (0.114)         (0.114)         (0.043)         (0.042)   
      Semi-urban / residential                   0.264***       -0.159***        0.028           0.150**         0.092           0.074           0.056*          0.044   
                                               (0.080)         (0.061)         (0.085)         (0.075)         (0.079)         (0.073)         (0.030)         (0.028)   
      Countryside                                0.167          -0.146**         0.244*         -0.082           0.058           0.037           0.022           0.004   
                                               (0.124)         (0.069)         (0.147)         (0.092)         (0.114)         (0.116)         (0.042)         (0.040)   
      Employed before pandemic                   0.341**         0.263***       -0.068           0.015           0.305***        0.166*          0.109***        0.119***
                                               (0.135)         (0.070)         (0.068)         (0.083)         (0.089)         (0.085)         (0.034)         (0.033)   
      Recent cases per capita                                                                                                                                   -6.841   
                                                                                                                                                               (7.091)   
      _cons                                      1.435***        0.338**         0.853***        1.532***        1.121***        1.096***        1.431***        1.318***
                                               (0.210)         (0.133)         (0.140)         (0.256)         (0.187)         (0.155)         (0.141)         (0.061)   
      Regional fixed effects                       Yes             Yes             Yes             Yes             Yes             Yes             Yes              No   
      Country fixed effects                         No              No              No              No              No              No             Yes             Yes   
      -------------------------------------------------------------------------------------------------------------------------------------------------------------------
      N                                            996            1013             962            1042            1016            1055            6084            6084   
      r2_a                                       0.065           0.038           0.019           0.015           0.016           0.021           0.116           0.111   
      -------------------------------------------------------------------------------------------------------------------------------------------------------------------
```

---

**REPLICATE TABLE A6**

```
      . qui eststo country1: reg bothered_social_distance i.age_large i.income female i.region i.current_living_area i.employment_status_pre_pandemic if country==1

      . qui eststo country2: reg bothered_social_distance i.age_large i.income female i.region i.current_living_area i.employment_status_pre_pandemic if country==2

      . qui eststo country3: reg bothered_social_distance i.age_large i.income female i.region i.current_living_area i.employment_status_pre_pandemic if country==3

      . qui eststo country4: reg bothered_social_distance i.age_large i.income female i.region i.current_living_area i.employment_status_pre_pandemic if country==4

      . qui eststo country5: reg bothered_social_distance i.age_large i.income female i.region i.current_living_area i.employment_status_pre_pandemic if country==5

      . qui eststo country6: reg bothered_social_distance i.age_large i.income female i.region i.current_living_area i.employment_status_pre_pandemic if country==6

      . qui eststo all: reg bothered_social_distance i.age_large i.income female i.country i.region i.current_living_area i.employment_status_pre_pandemic

      . qui eststo all2: reg bothered_social_distance i.age_large i.income female i.country i.current_living_area i.employment_status_pre_pandemic c.recent_cases
```

Table A6: Ordinary least squares for index of dissatisfaction with social distance

```
      . estout country1 country2 country3 country4 country5 country6 all all2, ///
               ce(b(star fmt(3) label("Coef")) se(par fmt(3))) nobaselevels label ///
               varwidth(35) stats(N r2_a, fmt(0 3)) title("TABLE A6") ///
               starl(* 0.10 ** 0.05 *** 0.01) ///
               keep(*.income_group *.age_large female *.current_living_area *.employment_status_pre_pandemic recent_cases_percapita _cons) ///
               drop(6.income_group) mlabels("China" "Japan" "Korea" "Italy" "UK"  "US") ///
               indicate("Regional fixed effects = *region" "Country fixed effects = *country") ///
               order(female *age_large *income_group *current_living_area *employment_status_pre_pandemic recent_cases_percapita)


      TABLE A6
      -------------------------------------------------------------------------------------------------------------------------------------------------------------------
                                                 China           Japan           Korea           Italy              UK              US             all            all2   
                                               Coef/se         Coef/se         Coef/se         Coef/se         Coef/se         Coef/se         Coef/se         Coef/se   
      -------------------------------------------------------------------------------------------------------------------------------------------------------------------
      Female                                    -0.253***        0.403***        0.161           0.042           0.098           0.107           0.097**         0.096** 
                                               (0.076)         (0.107)         (0.110)         (0.084)         (0.099)         (0.099)         (0.039)         (0.039)   
      Age 26-45                                 -0.214*         -0.150          -0.290           0.140          -0.276           0.058          -0.147**        -0.131** 
                                               (0.116)         (0.189)         (0.176)         (0.145)         (0.170)         (0.164)         (0.065)         (0.064)   
      Age 46-65                                 -0.279**        -0.275          -0.279          -0.009          -0.208          -0.006          -0.185***       -0.180***
                                               (0.126)         (0.188)         (0.179)         (0.146)         (0.172)         (0.162)         (0.065)         (0.064)   
      Age above 66                              -0.047          -0.636***       -0.706***       -0.100          -0.427**         0.092          -0.347***       -0.334***
                                               (0.165)         (0.221)         (0.206)         (0.174)         (0.211)         (0.186)         (0.077)         (0.076)   
      Second quintile                            0.341***        0.073           0.369**        -0.016           0.392**         0.270*          0.228***        0.235***
                                               (0.123)         (0.173)         (0.175)         (0.144)         (0.167)         (0.155)         (0.063)         (0.063)   
      Third quintile                             0.322***        0.064           0.585***        0.142           0.389**         0.227           0.263***        0.271***
                                               (0.125)         (0.173)         (0.168)         (0.138)         (0.164)         (0.153)         (0.062)         (0.061)   
      Fourth quintile                            0.290**         0.191           0.800***        0.327**         0.530***        0.319**         0.414***        0.420***
                                               (0.126)         (0.182)         (0.169)         (0.137)         (0.161)         (0.151)         (0.062)         (0.062)   
      Fifth quintile                             0.356***        0.367*          0.701***        0.396***        0.499***        0.266           0.424***        0.425***
                                               (0.136)         (0.189)         (0.181)         (0.153)         (0.165)         (0.165)         (0.066)         (0.065)   
      Semi-urban / residential                   0.135           0.039          -0.352**         0.142          -0.028           0.012           0.020           0.026   
                                               (0.098)         (0.130)         (0.158)         (0.099)         (0.114)         (0.106)         (0.047)         (0.044)   
      Countryside                               -0.014           0.066          -0.664**        -0.145           0.014           0.215          -0.008           0.011   
                                               (0.151)         (0.147)         (0.271)         (0.122)         (0.164)         (0.167)         (0.064)         (0.061)   
      Employed before pandemic                   0.913***        0.525***        0.289**         0.245**         0.156           0.579***        0.368***        0.376***
                                               (0.165)         (0.149)         (0.125)         (0.111)         (0.129)         (0.123)         (0.052)         (0.051)   
      Recent cases per capita                                                                                                                                   -2.817   
                                                                                                                                                              (10.877)   
      _cons                                     -1.332***       -0.495*         -0.467*         -0.115          -0.371          -0.563**        -0.977***       -0.395***
                                               (0.256)         (0.281)         (0.259)         (0.340)         (0.270)         (0.224)         (0.216)         (0.093)   
      Regional fixed effects                       Yes             Yes             Yes             Yes             Yes             Yes             Yes              No   
      Country fixed effects                         No              No              No              No              No              No             Yes             Yes   
      -------------------------------------------------------------------------------------------------------------------------------------------------------------------
      N                                            996            1013             962            1042            1016            1055            6084            6084   
      r2_a                                       0.103           0.059           0.054           0.043           0.043           0.036           0.045           0.042   
      -------------------------------------------------------------------------------------------------------------------------------------------------------------------
```

---

**REPLICATE TABLE A7**

```
      . import excel "data_for_replication/geo_distribution.xlsx", sheet("Sheet1") firstrow case(lower) clear
      (7 vars, 131 obs)

      . keep if area_survey !=""
      (9 observations deleted)

      . rename area_survey region 


      . drop g 

      . collapse (sum) population, by(region country)

      . save "data_for_replication/geo_distribution.dta", replace
      file data_for_replication/geo_distribution.dta saved

      . use "data_produced/clean_data", clear

      . sdecode country, replace


      . sdecode region, replace


      . replace region ="Kansai" if region=="Kansai "
      (0 real changes made)

      . replace region ="Fujian" if region=="Fujian "
      (0 real changes made)

      . replace region ="Gansu" if region=="Gansu "
      (0 real changes made)

      . merge m:m country region using "data_for_replication/geo_distribution.dta"

          Result                           # of obs.
          -----------------------------------------
          not matched                             0
          matched                             6,089  (_merge==3)
          -----------------------------------------

      . collapse (mean) population (count) observations=population, by(country region)

      . bysort country: egen country_pop=total(population)

      . bysort country: egen country_obs=total(observations)

      . gen pop_share = population/country_pop

      . gen sample_share = observations/country_obs

      . keep country region pop_share sample_share

      . format pop_share %9.3f

      . format sample_share %9.3f

      . sort country region
```

Table A7: Geographical Representation

```
      . list

           +--------------------------------------------------------------------+
           | country                               region   pop_sh~e   sample~e |
           |--------------------------------------------------------------------|
        1. |   China                                Anhui      0.053      0.054 |
        2. |   China                               Fujian      0.033      0.048 |
        3. |   China                                Gansu      0.022      0.008 |
        4. |   China                            Guangdong      0.095      0.153 |
        5. |   China                              Guizhou      0.030      0.010 |
           |--------------------------------------------------------------------|
        6. |   China                               Hainan      0.008      0.006 |
        7. |   China                                Hebei      0.063      0.092 |
        8. |   China                         Heilongjiang      0.032      0.029 |
        9. |   China                                Henan      0.080      0.047 |
       10. |   China                                Hubei      0.050      0.038 |
           |--------------------------------------------------------------------|
       11. |   China                                Hunan      0.058      0.032 |
       12. |   China                              Jiangsu      0.067      0.094 |
       13. |   China                              Jiangxi      0.039      0.027 |
       14. |   China                                Jilin      0.023      0.022 |
       15. |   China                             Liaoning      0.036      0.052 |
           |--------------------------------------------------------------------|
       16. |   China                              Qinghai      0.005      0.004 |
       17. |   China                              Shaanxi      0.032      0.027 |
       18. |   China                             Shandong      0.084      0.074 |
       19. |   China                               Shanxi      0.031      0.047 |
       20. |   China                              Sichuan      0.070      0.065 |
           |--------------------------------------------------------------------|
       21. |   China                               Yunnan      0.040      0.010 |
       22. |   China                             Zhejiang      0.048      0.058 |
       23. |   Italy                              Abruzzo      0.022      0.019 |
       24. |   Italy                           Basilicata      0.009      0.015 |
       25. |   Italy                             Calabria      0.032      0.021 |
           |--------------------------------------------------------------------|
       26. |   Italy                             Campania      0.096      0.090 |
       27. |   Italy                       Emilia-Romagna      0.074      0.072 |
       28. |   Italy                Friuli-Venezia Giulia      0.020      0.021 |
       29. |   Italy                                Lazio      0.097      0.116 |
       30. |   Italy                              Liguria      0.026      0.027 |
           |--------------------------------------------------------------------|
       31. |   Italy                            Lombardia      0.167      0.174 |
       32. |   Italy                               Marche      0.025      0.019 |
       33. |   Italy                               Molise      0.005      0.005 |
       34. |   Italy                             Piemonte      0.072      0.076 |
       35. |   Italy                               Puglia      0.067      0.078 |
           |--------------------------------------------------------------------|
       36. |   Italy                             Sardegna      0.027      0.044 |
       37. |   Italy                              Sicilia      0.083      0.063 |
       38. |   Italy                              Toscana      0.062      0.059 |
       39. |   Italy                  Trentino-Alto Adige      0.018      0.009 |
       40. |   Italy                               Umbria      0.015      0.008 |
           |--------------------------------------------------------------------|
       41. |   Italy                        Valle d'Aosta      0.002      0.003 |
       42. |   Italy                               Veneto      0.081      0.081 |
       43. |   Japan                                Chūbu      0.168      0.156 |
       44. |   Japan                              Chūgoku      0.058      0.055 |
       45. |   Japan                             Hokkaidō      0.042      0.044 |
           |--------------------------------------------------------------------|
       46. |   Japan                               Kansai      0.177      0.167 |
       47. |   Japan                                Kantō      0.343      0.421 |
       48. |   Japan                               Kyūshū      0.113      0.070 |
       49. |   Japan                              Shikoku      0.030      0.024 |
       50. |   Japan                               Tōhoku      0.069      0.063 |
           |--------------------------------------------------------------------|
       51. |   Korea                                Busan      0.066      0.086 |
       52. |   Korea                   Chung-cheong bukdo      0.031      0.025 |
       53. |   Korea                    Chungcheongnam-do      0.042      0.024 |
       54. |   Korea              Daegu Metropolitan City      0.047      0.059 |
       55. |   Korea                              Daejeon      0.029      0.030 |
           |--------------------------------------------------------------------|
       56. |   Korea                           Gangwon-do      0.029      0.028 |
       57. |   Korea                          Gyeonggi-do      0.283      0.239 |
       58. |   Korea                     Gyeongsangbuk-do      0.052      0.045 |
       59. |   Korea                     Gyeongsangnam-do      0.065      0.051 |
       60. |   Korea            Incheon Metropolitan City      0.057      0.056 |
           |--------------------------------------------------------------------|
       61. |   Korea                          Jeju Island      0.013      0.010 |
       62. |   Korea                         Jeollabuk do      0.035      0.028 |
       63. |   Korea                         Jeollanam-do      0.035      0.035 |
       64. |   Korea   Sejong Special Self-governing City      0.006      0.004 |
       65. |   Korea                                Seoul      0.187      0.268 |
           |--------------------------------------------------------------------|
       66. |   Korea              Ulsan Metropolitan City      0.022      0.011 |
       67. |      UK                        East Midlands      0.072      0.073 |
       68. |      UK                      East of England      0.093      0.080 |
       69. |      UK                       Greater London      0.134      0.156 |
       70. |      UK                           North East      0.040      0.050 |
           |--------------------------------------------------------------------|
       71. |      UK                           North West      0.110      0.094 |
       72. |      UK                     Northern Ireland      0.028      0.023 |
       73. |      UK                             Scotland      0.082      0.083 |
       74. |      UK                           South East      0.137      0.146 |
       75. |      UK                           South West      0.084      0.091 |
           |--------------------------------------------------------------------|
       76. |      UK                                Wales      0.047      0.045 |
       77. |      UK                        West Midlands      0.089      0.083 |
       78. |      UK             Yorkshire and the Humber      0.082      0.078 |
       79. |      US                           California      0.361      0.257 |
       80. |      US                              Florida      0.196      0.260 |
           |--------------------------------------------------------------------|
       81. |      US                             New York      0.178      0.287 |
       82. |      US                                Texas      0.265      0.196 |
           +--------------------------------------------------------------------+
```

---

**REPLICATE TABLES A9 AND A10**

```
      . use "data_produced/clean_data", clear

      . qui foreach var of var lost_job_atleast_temporarily started_teleworking fall_consumption neg_non_fin pos_non_fin {

      . mat table = lost_job_atleast_temporarily,started_teleworking,fall_consumption,neg_non_fin,pos_non_fin

      . clear

      . svmat table
      number of observations will be reset to 36
      Press any key to continue, or Break to abort
      number of observations (_N) was 0, now 36

      . foreach x of var * {
      . qui replace `x'=round(`x',0.001)
      . }

      . qui tostring *, replace format(%9.3f) force

      . forval n=1(1)15{
      . qui replace table`n' = "(" + table`n' + ")" if inlist(_n,2,4,6,8,10,12,14,16,18,20,22,24,26,28,30,32,34,36)
      . }

      . preserve 

      . keep table1 table4 table7 table10 table13

      . gen Country = ""
      (36 missing values generated)

      . loc j = 1

      . foreach state in China Japan Korea Italy UK US{
      .         qui replace Country = "`state'" in `j'
      .         loc j = `j' + 6
      . }

      . gen Prevalence = ""
      (36 missing values generated)

      . foreach num of numlist 1 7 13 19 25 31{
      .         loc j = `num'
      .         foreach name in All Low High{
      .                 qui replace Prevalence = "`name'" in `j'
      .                 loc j = `j' + 2
      .         }
      . }

      . rename table1 Lost_job


      . rename table4 Teleworking


      . rename table7 Spending_drop 


      . rename table10 Negative_well_being


      . rename table13 Positive_well_being


      . order Country Prevalence
```

Table A9: Mean Outcomes by Infection Prevalence Areas (standard errors in parentheses)

```
      . list, separator(6)

           +---------------------------------------------------------------------------+
           | Country   Preval~e   Lost_job   Telewo~g   Spendi~p   Negati~g   Positi~g |
           |---------------------------------------------------------------------------|
        1. |   China        All      0.432      0.517      0.563      1.673      1.445 |
        2. |                       (0.016)    (0.016)    (0.016)    (0.034)    (0.031) |
        3. |                Low      0.467      0.459      0.559      1.602      1.480 |
        4. |                       (0.025)    (0.025)    (0.025)    (0.055)    (0.050) |
        5. |               High      0.409      0.555      0.566      1.719      1.422 |
        6. |                       (0.020)    (0.020)    (0.020)    (0.043)    (0.039) |
           |---------------------------------------------------------------------------|
        7. |   Japan        All      0.145      0.188      0.308      1.229      0.596 |
        8. |                       (0.011)    (0.012)    (0.015)    (0.034)    (0.025) |
        9. |                Low      0.101      0.063      0.280      1.042      0.545 |
       10. |                       (0.022)    (0.018)    (0.033)    (0.074)    (0.058) |
       11. |               High      0.155      0.217      0.315      1.271      0.608 |
       12. |                       (0.013)    (0.014)    (0.016)    (0.038)    (0.027) |
           |---------------------------------------------------------------------------|
       13. |   Korea        All      0.250      0.147      0.490      1.410      0.952 |
       14. |                       (0.014)    (0.011)    (0.016)    (0.035)    (0.029) |
       15. |                Low      0.254      0.121      0.461      1.371      0.864 |
       16. |                       (0.026)    (0.020)    (0.030)    (0.065)    (0.054) |
       17. |               High      0.249      0.158      0.502      1.426      0.988 |
       18. |                       (0.017)    (0.014)    (0.019)    (0.041)    (0.034) |
           |---------------------------------------------------------------------------|
       19. |   Italy        All      0.369      0.261      0.566      1.640      1.606 |
       20. |                       (0.015)    (0.014)    (0.015)    (0.038)    (0.031) |
       21. |                Low      0.378      0.251      0.549      1.720      1.505 |
       22. |                       (0.022)    (0.020)    (0.023)    (0.056)    (0.045) |
       23. |               High      0.361      0.269      0.581      1.572      1.692 |
       24. |                       (0.020)    (0.019)    (0.021)    (0.051)    (0.042) |
           |---------------------------------------------------------------------------|
       25. |      UK        All      0.286      0.182      0.396      1.659      1.318 |
       26. |                       (0.014)    (0.012)    (0.015)    (0.043)    (0.034) |
       27. |                Low      0.268      0.157      0.396      1.553      1.316 |
       28. |                       (0.020)    (0.016)    (0.022)    (0.061)    (0.049) |
       29. |               High      0.304      0.206      0.395      1.761      1.320 |
       30. |                       (0.020)    (0.018)    (0.021)    (0.061)    (0.046) |
           |---------------------------------------------------------------------------|
       31. |      US        All      0.323      0.231      0.431      1.693      1.177 |
       32. |                       (0.014)    (0.013)    (0.015)    (0.042)    (0.032) |
       33. |                Low      0.305      0.184      0.439      1.655      1.232 |
       34. |                       (0.021)    (0.018)    (0.023)    (0.063)    (0.048) |
       35. |               High      0.338      0.270      0.425      1.724      1.132 |
       36. |                       (0.020)    (0.019)    (0.021)    (0.056)    (0.044) |
           +---------------------------------------------------------------------------+

      . restore
```

---

```
      . preserve 

      . drop table1 table4 table7 table10 table13

      . gen Country = ""
      (36 missing values generated)

      . loc j = 1

      . foreach state in China Japan Korea Italy UK US{
      .         qui replace Country = "`state'" in `j'
      .         loc j = `j' + 6
      . }

      . gen Prevalence = ""
      (36 missing values generated)

      . foreach num of numlist 1 7 13 19 25 31{
      .         loc j = `num'
      .         foreach name in All Low High{
      .                 qui replace Prevalence = "`name'" in `j'
      .                 loc j = `j' + 2
      .         }
      . }

      . rename table2 Lost_job_Age


      . rename table5 Teleworking_Age


      . rename table8 Spending_drop_Age


      . rename table11 Negative_well_being_Age


      . rename table14 Positive_well_being_Age


      . rename table3 Lost_job_Income


      . rename table6 Teleworking_Income


      . rename table9 Spending_drop_Income


      . rename table12 Negative_well_being_Income


      . rename table15 Positive_well_being_Income


      . order Country Prevalence
```

Table A10: Linear Age and Income Gradients by Infection Prevalence Areas (p-values in parentheses)

```
      . list, separator(6)

           +----------------------------------------------------------------------------------------------------------------------------------+
           | Country   Preval~e   Lost_~ge   Lost_~me   Telew~ge   Telew~me   Spend~ge   Spend~me   Negat~ge   Negat~me   Posit~ge   Posit~me |
           |----------------------------------------------------------------------------------------------------------------------------------|
        1. |   China        All      0.056     -0.069     -0.172      0.018     -0.085     -0.031     -0.194      0.016     -0.203      0.015 |
        2. |                       (0.004)    (0.000)    (0.000)    (0.121)    (0.000)    (0.015)    (0.000)    (0.538)    (0.000)    (0.532) |
        3. |                Low      0.110     -0.052     -0.212      0.042     -0.068      0.005     -0.188      0.106     -0.247      0.099 |
        4. |                       (0.002)    (0.006)    (0.000)    (0.018)    (0.065)    (0.816)    (0.014)    (0.012)    (0.000)    (0.009) |
        5. |               High      0.027     -0.080     -0.153     -0.002     -0.092     -0.053     -0.195     -0.046     -0.180     -0.045 |
        6. |                       (0.251)    (0.000)    (0.000)    (0.890)    (0.000)    (0.001)    (0.000)    (0.164)    (0.000)    (0.152) |
           |----------------------------------------------------------------------------------------------------------------------------------|
        7. |   Japan        All     -0.012     -0.032     -0.053      0.065     -0.053     -0.009     -0.203      0.009      0.036      0.019 |
        8. |                       (0.397)    (0.000)    (0.000)    (0.000)    (0.004)    (0.461)    (0.000)    (0.747)    (0.257)    (0.343) |
        9. |                Low      0.033     -0.027     -0.017      0.036     -0.043     -0.010     -0.236      0.084     -0.157      0.037 |
       10. |                       (0.281)    (0.196)    (0.498)    (0.039)    (0.346)    (0.757)    (0.017)    (0.214)    (0.045)    (0.490) |
       11. |               High     -0.020     -0.034     -0.058      0.069     -0.055     -0.008     -0.196     -0.003      0.073      0.019 |
       12. |                       (0.188)    (0.000)    (0.001)    (0.000)    (0.007)    (0.531)    (0.000)    (0.909)    (0.034)    (0.379) |
           |----------------------------------------------------------------------------------------------------------------------------------|
       13. |   Korea        All      0.002     -0.065     -0.042      0.034      0.007     -0.015     -0.165     -0.016     -0.118      0.010 |
       14. |                       (0.877)    (0.000)    (0.002)    (0.000)    (0.732)    (0.230)    (0.000)    (0.557)    (0.000)    (0.659) |
       15. |                Low      0.042     -0.066     -0.020      0.035     -0.005     -0.008     -0.160      0.067     -0.131     -0.042 |
       16. |                       (0.121)    (0.000)    (0.373)    (0.025)    (0.880)    (0.739)    (0.032)    (0.179)    (0.043)    (0.337) |
       17. |               High     -0.015     -0.065     -0.049      0.034      0.009     -0.020     -0.158     -0.051     -0.114      0.027 |
       18. |                       (0.414)    (0.000)    (0.003)    (0.001)    (0.698)    (0.177)    (0.001)    (0.099)    (0.004)    (0.293) |
           |----------------------------------------------------------------------------------------------------------------------------------|
       19. |   Italy        All     -0.014     -0.066     -0.023      0.071      0.013     -0.010     -0.262     -0.033      0.047      0.051 |
       20. |                       (0.398)    (0.000)    (0.152)    (0.000)    (0.517)    (0.434)    (0.000)    (0.265)    (0.239)    (0.046) |
       21. |                Low      0.005     -0.072     -0.024      0.067      0.007      0.007     -0.254     -0.026      0.061      0.058 |
       22. |                       (0.813)    (0.000)    (0.308)    (0.000)    (0.800)    (0.690)    (0.000)    (0.561)    (0.278)    (0.116) |
       23. |               High     -0.037     -0.065     -0.020      0.080      0.017     -0.028     -0.276     -0.036      0.037      0.038 |
       24. |                       (0.109)    (0.000)    (0.356)    (0.000)    (0.540)    (0.117)    (0.000)    (0.371)    (0.504)    (0.295) |
           |----------------------------------------------------------------------------------------------------------------------------------|
       25. |      UK        All     -0.044     -0.053     -0.040      0.044      0.008      0.021     -0.340     -0.080      0.133      0.001 |
       26. |                       (0.008)    (0.000)    (0.007)    (0.000)    (0.665)    (0.058)    (0.000)    (0.011)    (0.003)    (0.957) |
       27. |                Low     -0.031     -0.040     -0.015      0.051      0.033      0.011     -0.263     -0.049      0.178      0.064 |
       28. |                       (0.188)    (0.004)    (0.460)    (0.000)    (0.227)    (0.479)    (0.001)    (0.278)    (0.006)    (0.092) |
       29. |               High     -0.063     -0.066     -0.062      0.040     -0.022      0.031     -0.412     -0.106      0.088     -0.056 |
       30. |                       (0.008)    (0.000)    (0.006)    (0.002)    (0.423)    (0.051)    (0.000)    (0.016)    (0.157)    (0.113) |
           |----------------------------------------------------------------------------------------------------------------------------------|
       31. |      US        All     -0.029     -0.039     -0.029      0.075     -0.018      0.043     -0.373     -0.007     -0.060      0.014 |
       32. |                       (0.062)    (0.000)    (0.041)    (0.000)    (0.333)    (0.000)    (0.000)    (0.830)    (0.140)    (0.587) |
       33. |                Low     -0.046     -0.041     -0.023      0.067     -0.018      0.048     -0.384     -0.012     -0.028     -0.041 |
       34. |                       (0.036)    (0.005)    (0.230)    (0.000)    (0.513)    (0.006)    (0.000)    (0.807)    (0.630)    (0.285) |
       35. |               High     -0.015     -0.037     -0.031      0.081     -0.016      0.038     -0.363     -0.001     -0.080      0.056 |
       36. |                       (0.488)    (0.005)    (0.120)    (0.000)    (0.533)    (0.014)    (0.000)    (0.985)    (0.158)    (0.104) |
           +----------------------------------------------------------------------------------------------------------------------------------+

      . restore
```

---
