# Supplementary material for: Unequal consequences of Covid 19: representative evidence from six countries
Source: Rev Econ Househ. 2021 Apr 7;19(3):769–83. doi: 10.1007/s11150-021-09560-z (PMC8025452; doi:10.1007/s11150-021-09560-z)
Supplement: Supplementary file 2 — Supplementary figures [file 11150_2021_9560_MOESM2_ESM.pdf]

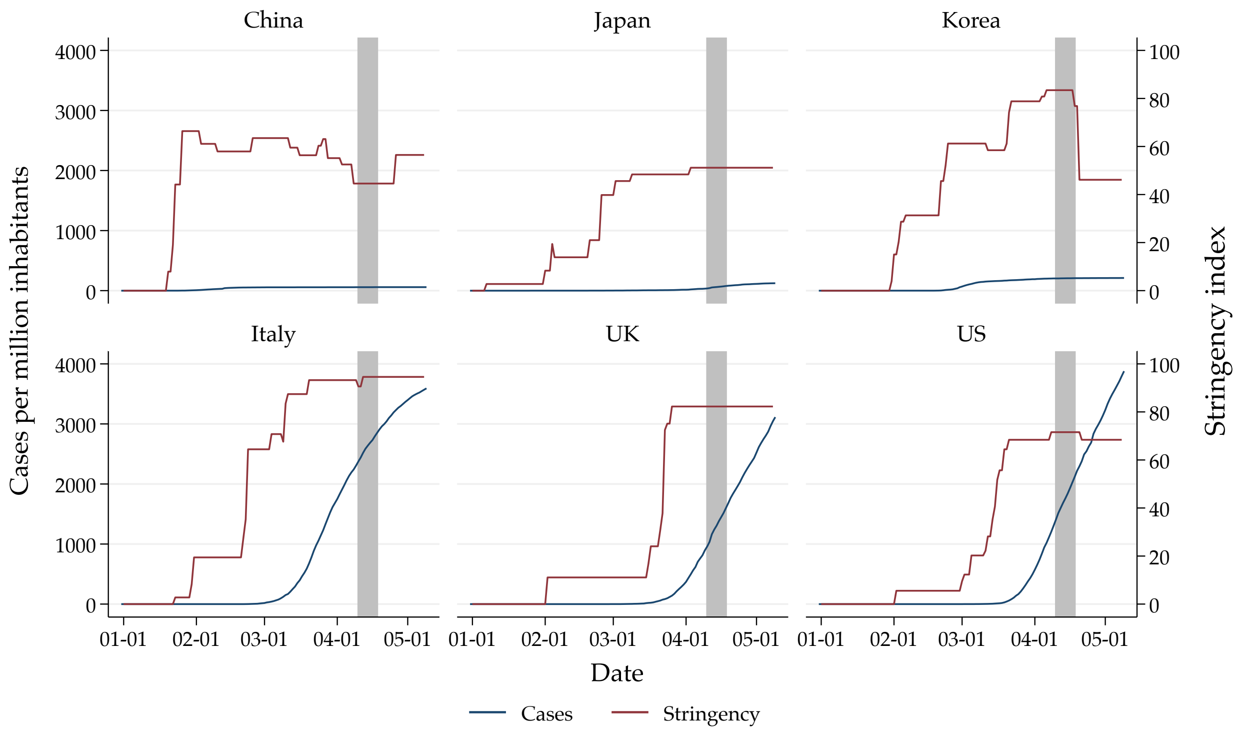

Note: The gray bar represents the third week of April 2020, in which the survey was conducted. Source: Hale et al. (2020).

Figure A1: Time series of the number of confirmed cases and stringency index of government responses

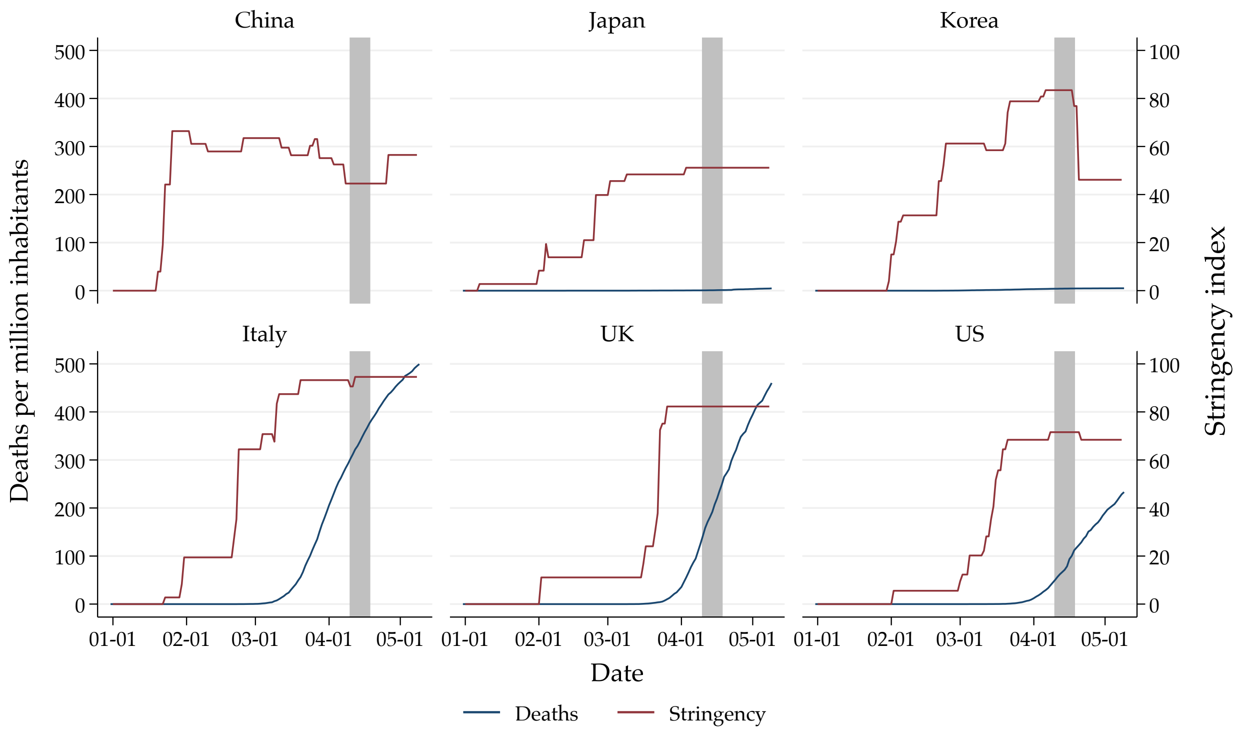

Note: The gray bar represents the third week of April 2020, in which the survey was conducted. Data on deaths from China is not available. Source: Hale et al. (2020).

Figure A2: Time series of the number of Covid-19 related deaths and stringency index of government responses
